# Supervised analysis of alternative polyadenylation from single-cell and spatial transcriptomics data with spvAPA

**DOI:** 10.1093/bib/bbae720

**Published:** 2025-01-11

**Authors:** Qinglong Zhang, Liping Kang, Haoran Yang, Fei Liu, Xiaohui Wu

**Affiliations:** Cancer Institute, Suzhou Medical College, Soochow University, NO. 199 Ren-ai Road, SIP, Suzhou 215000, China; Cancer Institute, Suzhou Medical College, Soochow University, NO. 199 Ren-ai Road, SIP, Suzhou 215000, China; Cancer Institute, Suzhou Medical College, Soochow University, NO. 199 Ren-ai Road, SIP, Suzhou 215000, China; Cancer Institute, Suzhou Medical College, Soochow University, NO. 199 Ren-ai Road, SIP, Suzhou 215000, China; Cancer Institute, Suzhou Medical College, Soochow University, NO. 199 Ren-ai Road, SIP, Suzhou 215000, China; Jiangsu Key Laboratory of Infection and Immunity, Soochow University, NO. 199 Ren-ai Road, SIP, Suzhou 215000, China

**Keywords:** alternative polyadenylation, single-cell RNA-seq, spatial transcriptomics, supervised analysis, visualization

## Abstract

Alternative polyadenylation (APA) is an important driver of transcriptome diversity that generates messenger RNA isoforms with distinct 3′ ends. The rapid development of single-cell and spatial transcriptomic technologies opened up new opportunities for exploring APA data to discover hidden cell subpopulations invisible in conventional gene expression analysis. However, conventional gene-level analysis tools are not fully applicable to APA data, and commonly used unsupervised dimensionality reduction methods often disregard experimentally derived annotations such as cell type identities. Here, we proposed a supervised analytical framework termed spvAPA, specifically used for APA analysis from both single-cell and spatial transcriptomics data. First, an iterative imputation method based on weighted nearest neighbor was designed to recover missing APA signatures, by integrating both gene expression and APA modalities. Second, a supervised feature selection method based on sparse partial least squares discriminant analysis was devised to identify APA features distinguishing cell types or spatial morphologies. Additionally, spvAPA improves the visualization of high-dimensional data for discovering novel cell subtypes, which considers APA features and dual modalities of gene expression and APA. Evaluations across nine single-cell and spatial transcriptomics datasets demonstrate the effectiveness and applicability of spvAPA. spvAPA is available at https://github.com/BMILAB/spvAPA.

## Introduction

Alternative polyadenylation (APA) is an essential post-transcriptional modification during messenger RNA (mRNA) maturation in eukaryotic cells [1], which generates multiple transcript isoforms with distinct 3′ ends via selective choice of different polyadenylation [poly(A)] sites. APA contributes greatly to the complexity of the transcriptome and the diversity of the proteome. APA is highly tissue specific and is dynamically regulated under various environmental conditions, cell types, and states [2–6]. APA also plays an important role in various biological processes, including cell proliferation, differentiation, neurological diseases, and tumorigenesis [7].

Currently, many sequencing strategies targeting 3′ ends of the transcripts have been developed for single-cell transcriptomics (e.g. 10x Chromium [8], Drop-seq [9], and mcSCRB-seq [10]) or spatial transcriptomics (e.g. 10x Visium [11]). These emerging technologies have also given rise to innovative bioinformatics analysis methods that enable the study of gene expression regulation at the transcript level rather than the conventional gene level [12, 13]. The transcript-level analysis has unveiled novel cell subtypes and disease-associated genes that are not discernible through traditional gene expression profiling alone [13–15]. A variety of tools, such as Sierra [12] and scAPAtrap [13], are available for identifying and quantifying poly(A) sites from single-cell RNA-seq (scRNA-seq) data. Similar to the gene–cell expression matrix obtained from scRNA-seq, an APA usage matrix ($\varnothing$), with each row denoting an APA gene and each column denoting a cell, can be obtained after quantifying genome-wide poly(A) sites. The $\varnothing$ matrix usually records the relative usage (denoted as *φ*) of the proximal or distal poly(A) site in the 3′ untranslated region (3′ UTR) of APA genes. However, due to the high dropout rates inherent in scRNA-seq, the $\varnothing$ matrix suffers from a considerably higher degree of sparsity than the gene–cell expression matrix, posing substantial challenges to transcriptome analysis from the APA layer. Moreover, unlike the gene–cell/spot expression matrix that consists of count values, the $\varnothing$ matrix typically holds ratios between 0 and 1, which fails to satisfy the underlying data distribution assumptions required by many gene-level analysis methodologies.

Currently, only a few tools have been developed for APA analysis from single-cell and/or spatial transcriptomics data, including scDaPars [15] and stAPAminer [16]. scDaPars, an extension of DaPars [17] that was originally designed for bulk RNA-seq, identifies poly(a) sites at the single-cell level and imputes missing entries in the $\varnothing$ matrix. Stapaminer was designed for identifying spatially variable APA genes from spatial transcriptomics data, wherein missing values were estimated by considering neighboring spots determined by gene expression profiles. However, both methods solely rely on data from a single modality (APA or gene expression) for recovering missing entries, overlooking the complementary nature between the two modalities. Moreover, these methods used existing gene-level methods for count data to identify differentially expressed genes (DEGs) or spatially variable genes (SVGs), which are not fully applicable to the $\varnothing$ matrix typically holding ratio values. For example, spark [18], used in stapaminer for identifying SVGs, expects the input count data to fit a Poisson distribution. In addition, single-cell or spatial transcriptomics data are high dimensional, commonly used unsupervised computational methods such as uniform manifold approximation and projection (UMAP) have been widely used for dimensional reduction and visualization [19]. Although these algorithms are powerful, they may not constantly be the best option for a specific dataset, depending on the biological questions and analytical objectives [20]. Moreover, although these unsupervised methods can construct an unbiased manifold representation from the data, they disregard experimentally derived, biologically meaningful annotations, such as cell type identities, tissue morphological annotations, or differentiation stages. These annotations carry critical metadata about the underlying drivers of biological variation and could potentially be harnessed to improve the interpretability and accuracy of data analysis if properly incorporated into the dimensionality reduction process [21]

In this study, we proposed a supervised analytical framework termed spvAPA specifically designed for APA analysis from both single-cell and spatial transcriptomics data. Firstly, we designed an iterative imputation method for imputing missing entries in the $\varnothing$ matrix. Secondly, a supervised feature selection method based on sparse partial least squares discriminant analysis (sPLS-DA) is devised to identify APA features from scRNA-seq and spatial transcriptomics data. Additionally, spvAPA integrates a flexible visualization module that considers both the selected features and the dual modalities of gene expression and APA, thereby enhancing the visualization of high-dimensional scRNA-seq or spatial transcriptomics data. Evaluations across nine datasets demonstrated the applicability and effectiveness of spvAPA.

## Materials and methods

**Figure 1:**
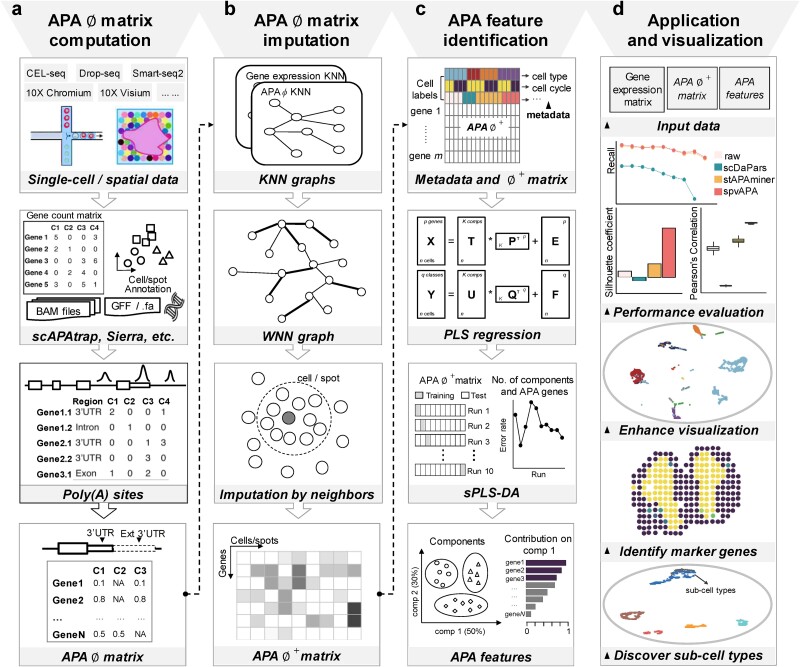
Schema of spvAPA. (a) The $\varnothing$ matrix computation module generates the raw APA $\varnothing$matrix from single-cell or spatial transcriptomics data. (b) The APA signature imputation module recovers missing entries in the APA $\varnothing$matrix using the WNN-based method. (c) The supervised feature selection module identifies APA features from the APA ${\varnothing}^{+}$matrix based on sPLS-DA that considers prior cell annotations. (d) The visualization module enhances dimensional reduction and visualization by integrating multimodal data for discovering subcell types and marker genes. WNN, weighted nearest neighbor; sPLS-DA, sparse partial least squares discriminant analysis.

spvAPA is a supervised analytical framework tailored for APA analysis from single-cell and spatial transcriptomics data, which consists of four modules: $\varnothing$ matrix computation module, APA signature imputation module, supervised feature selection module, and visualization module (Fig. 1). The $\varnothing$ matrix computation module (Fig. 1a) is responsible for generating the APA usage matrix. Genome-wide poly(A) sites were identified and quantified from single-cell and spatial transcriptomics data, and genes with at least two poly(A) sites in the 3′ UTR (called 3′ UTR-APA genes) were retained. Then, the APA usage ($\varphi$) for each 3′ UTR-APA gene, represented by the relative usage of distal poly(A) site (RUD), was calculated to construct the $\varnothing$ matrix. Next, by integrating the $\varnothing$ matrix and the gene–cell expression matrix, the APA signature imputation module recovers missing values in the ∅ matrix using an iterative method based on weighted nearest neighbor (WNN) [22] (Fig. 1b). Further, the supervised feature selection module (Fig. 1c) applies sPLS-DA to the matrix after imputation (called ${\varnothing}^{+}$) in a supervised learning fashion. It considers metadata (e.g. cell or spot label) to identify APA features that distinguish different cell types or spatial domains. Lastly, the visualization module (Fig. 1d) integrates selected APA features and both gene expression and APA modalities for visualizing high-dimensional data. This allows for enhanced visualization of single-cell or spatial transcriptomics data, revealing potentially hidden cell subpopulations that might not be discovered solely through the gene expression profile.

A total of nine single-cell and spatial transcriptomics datasets were analyzed in this study (Supplementary Table 1), including two datasets of mouse olfactory bulb (sc-MOB1 and sc-MOB2) [23], a mouse spermatogenesis dataset [24], an HESC dataset [25], two datasets of human peripheral blood mononuclear cells (pbmc4k and pbmc8k), and three tissue sections of st-MOB analyzed in previous studies [7, 16, 26–29] (st-MOB5, st-MOB11, and st-MOB12). The single-cell datasets were all collected from whole cells rather than nuclei. Pearson’s correlation coefficients (PCCs) and silhouette coefficient (SC) score [30] were used to evaluate the performance of the imputation methods. More details are described in the supplementary text.

## Results

### spvAPA effectively recovers alternative polyadenylation signatures in single-cell and spatial transcriptomics data

**Figure 2:**
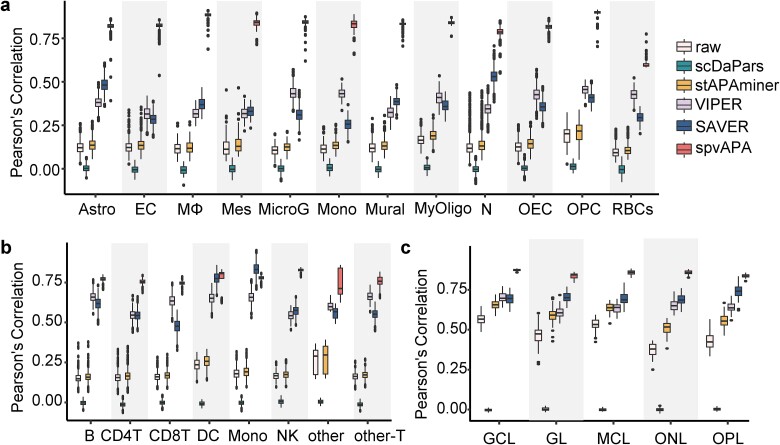
Pearson’s correlations estimated using imputed ${\varnothing}^{+}$ matrices by different methods and the raw $\varnothing$ matrix for SC-MOB1 (a), PBMC4K (b), and ST-MOB11 (c). For each dataset, the Pearson’s correlation of the APA profile of each cell/spot in each cell type/layer with the average APA profile of the raw data in the respective cell type/layer were calculated.

The spvAPA framework includes an unsupervised WNN-based imputation module for recovering the highly sparse APA matrix $\varnothing$. Here, we evaluated the performance of spvAPA’s imputation method using seven single-cell and spatial transcriptomics datasets (Supplementary Table 1) and compared spvAPA against two existing APA signature imputation methods, namely, scDaPars [15] and stAPAminer [16]. We also compared spvAPA with another two tools for imputing single-cell gene expression data, SAVER [31] and VIPER [32]. Since the APA profile of the imputed matrix ${\varnothing}^{+}$ should closely resemble that of the original matrix $\varnothing$, we used the PCC to evaluate the correlation between the two matrices (see Materials and Methods in Supplementary Text). For all the seven datasets, the cell–cell or spot–spot PCC values after imputation by spvAPA were greatly improved (Fig. 2 and Supplementary Fig. 1). After imputation using stAPAminer, an improvement in PCC was also observed. In contrast, scDaPars performed the worst, with the PCC value close to zero and, in some cases, even lower than the PCC of the raw data. Surprisingly, SAVER or VIPER, originally designed for imputing gene expression data, performed even better than stAPAminer or scDaPars. Overall, spvAPA performs the best among all tools, achieving much higher PCC values than other tools.

Next, we further evaluated the robustness and effectiveness of the WNN module from different perspectives. First, we compared the WNN module with the latest deep learning model called JAMIE (Joint Variational Autoencoders for Multimodal Imputation and Embedding) [33]. Results across the seven datasets showed that the WNN outperformed JAMIE in imputing APA signatures (Supplementary Fig. 2). Second, we evaluated the imputation effect of the WNN module on imbalanced datasets. The results suggested that the performance improvement of rare cell types after imputation is lower than that of normal cell types, but imputation always contributes to performance improvement (Supplementary Fig. 3). Moreover, we examined the impact of the parameter of the WNN module that determines the number of nearing neighbors ($k$) on APA matrix imputation and APA feature selection. Results demonstrated the robustness of the WNN method to different $k$ values (Supplementary Fig. 4). In addition, we evaluated the computational efficiency of the WNN method on datasets with a different number of genes and cells, and results demonstrated its scalability (Supplementary Fig. 5). The details of these results are provided in Supplementary Text Note 1.

**Figure 3:**
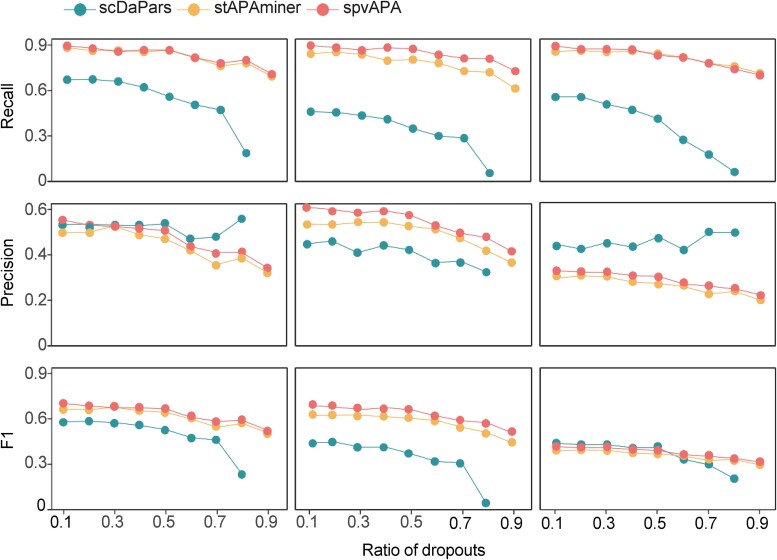
Evaluation of different methods in recovering genes with differentially used APA sites (DEAPA genes) using the mouse spermatogenesis dataset. DEAPA genes among each pair of the three developmental states during mouse spermatogenesis, including SCs, RSs, and ESs, were calculated. DEAPA genes from the raw matrix $\varnothing$ without masking were used as the reference. Nonmissing entries in the $\varnothing$ matrix were randomly masked to construct matrices with higher dropout rate from 10% to 90%. Three imputation methods were then applied to these matrices for recovering APA signatures, and DEAPA genes were obtained.

Next, we explored spvAPA’s capability in recovering genes with differentially used APA sites (called DEAPA genes). We used the mouse spermatogenesis dataset [24] for evaluation as distinct APA dynamics have been revealed during the three spermatic states, spermatocytes (SCs), round spermatids (RSs), and elongating spermatids (ESs) [13, 14, 34–36]. First, we identified DEAPA genes from the matrix $\varnothing$ (before imputation) as the reference. We applied the Wilcoxon test to test the significance of differences on $\mathrm{\varphi}$ values of the matrix $\varnothing$ for each APA gene between each pair of cell types and obtained DEAPA genes among the three stages. For instance, we identified 684 DEAPA genes between SC and ES, with 592 DEAPA genes with longer 3′ UTR in SC and only 92 in ES (Supplementary Fig. 6), aligning with previous studies that reported a shortening of 3′ UTR length in sperm cells as development progresses (SC → RS → ES) [13, 14, 34–36]. Next, we randomly masked 10%–90% nonmissing entries in the $\varnothing$ matrix to construct a matrix ${\varnothing}^{-}$ with a higher dropout rate. Accordingly, the same entries in the corresponding gene expression matrix $G$ were masked. This allowed us to use the original matrix $\varnothing$ as the gold standard for assessing the efficacy of imputation methods. After imputing the matrix ${\varnothing}^{-}$ with different imputation methods, DEAPA genes were identified. The recovery effect of DEAPA genes by different methods was evaluated by precision, recall, and F1 score (Fig. 3). The recall values of stAPAminer and spvAPA were comparable, with spvAPA exhibiting a marginally higher recall than stAPAminer and both demonstrating much higher performance than scDaPars in this regard. In terms of precision, spvAPA surpassed stAPAminer, but it had a mixed record against scDaPars. Using F1 score as a combined measure of precision and recall, spvAPA consistently outperformed stAPAminer and showed a notably superior performance over scDaPars in the majority of cases. These results suggested the high performance of spvAPA in effective recovering of APA signatures from data with high dropout rates.

### spvAPA enhances dimensionality reduction and visualization for single-cell and spatial transcriptomics data

**Figure 4:**
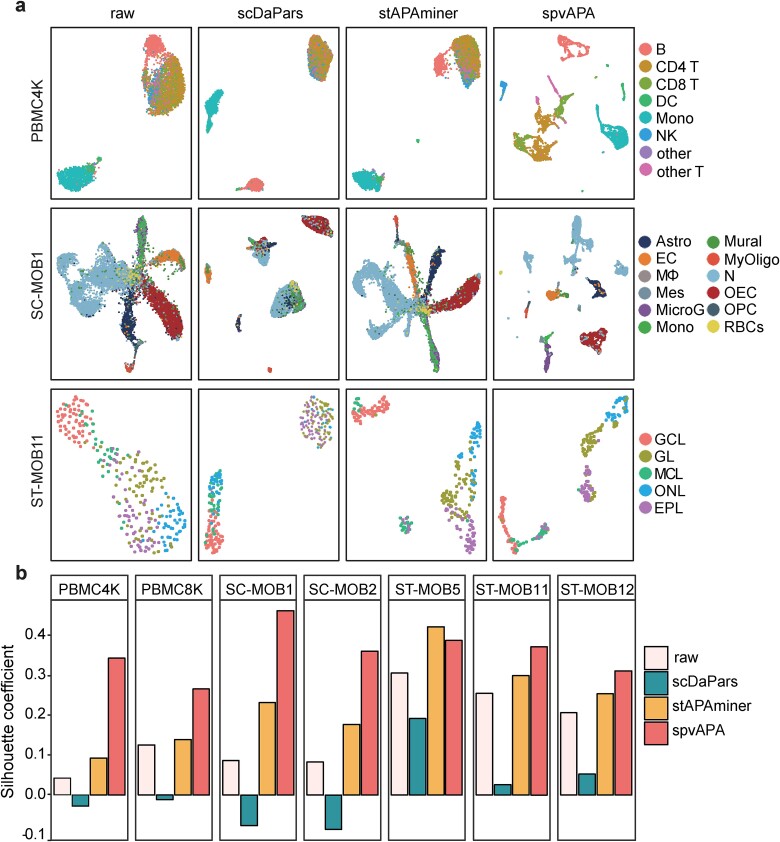
Dimensionality reduction and visualization for single-cell and spatial transcriptomics data. (a) UMAP visualization for the raw $\varnothing$ matrix or imputed ${\varnothing}^{+}$ matrices by different methods for PBMC4K, SC-MOB1, and ST-MOB11. For each matrix, normalization and dimensionality reduction with PCA were first performed by Seurat before UMAP visualization. (b) Evaluation of different methods in distinguishing cell types for different datasets using SC.

Having demonstrated that spvAPA can effectively recover the APA profile, we then examined whether the recovered APA profile can enhance the visualization of single-cell data to more clearly distinguish different cell types. Across all seven datasets, the UMAP visualization of the ${\varnothing}^{+}$ matrices derived from spvAPA showed superior performance compared to both the original $\varnothing$ matrix and the imputed ${\varnothing}^{+}$ matrices from stAPAminer and scDaPars, indicating notably enhanced differentiation among distinct cell types (Fig. 4a and Supplementary Fig. 7). Particularly, in complex tissue samples like PBMC4K and SC-MOB1 (Fig. 4a), the visualization of the ${\varnothing}^{+}$ matrix obtained by spvAPA clearly differentiates all cell types. In contrast, for PBMC4K, the visualization of the matrix ${\varnothing}^{+}$ obtained by stAPAminer or scDaPars only separated B cells and mononuclear cells (Mono) but failed to distinguish other cell types. For SC-MOB1, the visualization of the original $\varnothing$ matrix or the ${\varnothing}^{+}$ matrix obtained by stAPAminer or scDaPars failed to separate almost all cell types.

Next, we used the SC metric to compare the effectiveness of ${\varnothing}^{+}$ matrices obtained by different methods in distinguishing different cell types. Across all seven datasets, except for st-MOB5, SC scores obtained by spvAPA were noticeably higher than those obtained by other methods or the original matrix $\varnothing$ (Fig. 4b). For st-mob5, the SC score achieved with spvAPA was only marginally lower than that of stapaminer (spvAPA = 0.389; stAPAminer = 0.423), yet still much higher than the SC score obtained by scDaPars (0.192). These results, as evidenced by evaluations across diverse scRNA-seq and spatial transcriptomics datasets, demonstrated that restoring the highly sparse APA profile via spvAPA enhanced visualization and enabled more accurate discrimination between different cell types and spatial domains.

### spvAPA discovers subcell types from scRNA-seq data

**Figure 5:**
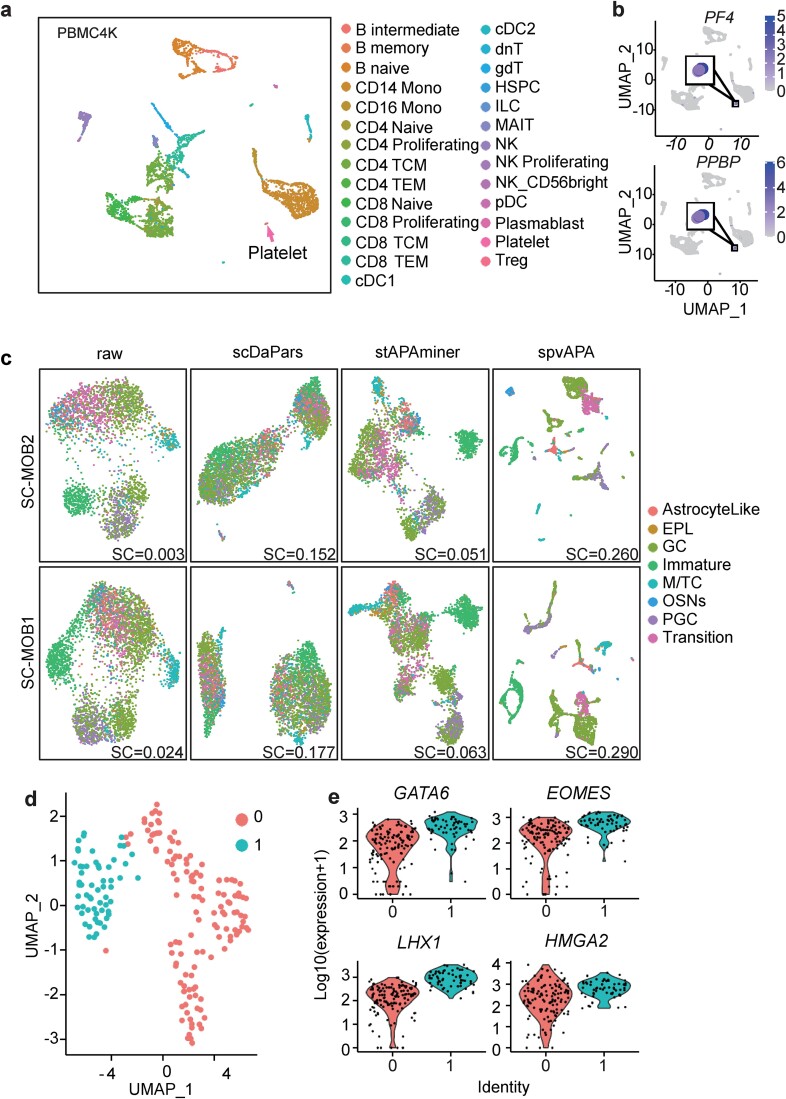
Discovery of subcell types from scRNA-seq data with spvAPA. (a) UMAP plot showing a small cluster of platelets in PBMC4K. (b) UMAP plot showing the expression of two platelet-specific marker genes, *PF4* and *PPBP*. (c) UMAP visualization of neuronal subtypes using the raw $\varnothing$ matrix or imputed ${\varnothing}^{+}$ matrices by different methods for SC-MOB1 and SC-MOB2. The corresponding SC score was shown on each plot. d UMAP plot using the matrix ${\varnothing}^{+}$ generated by spvAPA shows that human embryonic stem cells differentiated for 96 h were further divided into two subclusters. (e) Expressions of four marker genes related to endoderm development in the two subclusters.

In the above visualization of the PBMC4K and PBMC8K data, we observed some small clusters containing very few single cells in the UMAP plots (Fig. 4a and Supplementary Fig. 7). Subsequently, we delved into the potential of spvAPA in discovering subcell types. We obtained additional annotation of subcell types using Azimuth [22] and found that a small cluster of cells in PBMC4K and PBMC8K were annotated as platelet (Fig. 5a and Supplementary Fig. 8a). Platelets are diminutive cellular fragments derived from megakaryocytes within the bone marrow. Within the milieu of PBMC samples, platelets are a scant fraction that have not been fully segregated, rendering their recognition challenging [22]. Two platelet-specific marker genes, *PF4* and *PPBP*, were universally highly expressed in the platelet cluster (Fig. 5b and Supplementary Fig. 8b), indicating that the small cluster exclusively discovered by spvAPA indeed is platelet. In contrast, using the original matrix $\varnothing$ or the matrix ${\varnothing}^{+}$ obtained by stAPAminer or scDaPars failed to distinguish platelets from other cells (Fig. 4a). Next, we proceeded to visualize another highly heterogeneous dataset, SC-MOB, to examine whether neuronal subtypes can be identified. The visualization of the original $\varnothing$ matrix or the ${\varnothing}^{+}$ matrices obtained through stAPAminer or scDaPars only distinguished one to two neuronal subtypes (Fig. 5c). In contrast, the visualization based on spvAPA-derived ${\varnothing}^{+}$ matrix revealed a clear separation of most neuronal subtypes. Moreover, SC values obtained by spvAPA were much higher than those from the original $\varnothing$ matrix and the ${\varnothing}^{+}$ matrices obtained from stAPAminer or scDaPars.

APA patterns are globally regulated and play a crucial role in cell differentiation. During embryonic development, the usage of proximal poly(a) site gradually decreases [6, 37]. Gao *et al.* [15] applied scdapars to the human embryonic stem cell data to obtain the matrix ${\varnothing}^{+}$, and identified a new cell subcell type invisible to conventional gene expression analysis. Next, we explored whether the matrix ${\varnothing}^{+}$ generated by spvAPA could aid in discovering novel subcell types. Initially, by applying spvAPA to impute the matrix ∅ of the HESC data and visualizing through UMAP, the imputed matrix ${\varnothing}^{+}$ was found to separate cells of various differentiation times (Supplementary Fig. 9a). Especially, in Gao *et al.* [15] who also analyzed the HESC data, cells differentiated for 72 and 96 h could not be distinguished, and cells differentiated for 12 and 24 h had low separability (Supplementary Fig. 9b). In contrast, the matrix ${\varnothing}^{+}$ generated by spvAPA clearly distinguished cells from different differentiation times. Using Louvain clustering, cells differentiated for 96 h were further divided into two subclusters, Subcluster 0 and Subcluster 1 (Fig. 5d). To verify that the identified subclusters represent different stages of differentiation, we examined four marker genes related to endoderm development: *gata6*, *eomes*, *lhx1*, and *hmga2*. *Gata6* and *eomes* were expressed higher in the subcluster with a higher degree of differentiation [25]. *lhx1* is crucial for kidney development [38]; *hmga2* is essential for epithelial differentiation during embryonic lung development [39]. These marker genes were more highly expressed in Subcluster 1, suggesting that Subcluster 1 was more differentiated than Subgroup 0 (Fig. 5e). These results demonstrated that the APA information restored by spvAPA could effectively discover subcell types and separate complex cell types invisible to conventional gene expression analysis.

### spvAPA identifies alternative polyadenylation features from scRNA-seq data and enhances visualization

**Figure 6:**
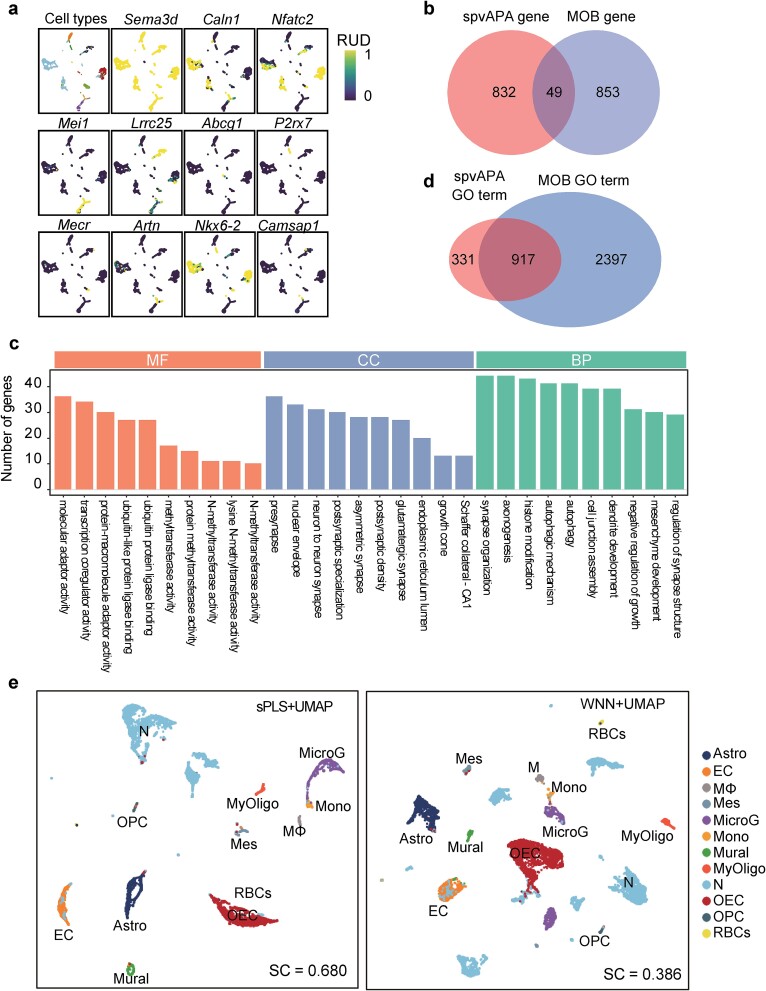
spvAPA identifies APA features from scRNA-seq data and enhances visualization. (a) Distribution of single-cell RUD scores of genes with the highest contribution in each component. (b) Venn diagram showing the overlap of APA features identified by spvAPA in SC-MOB1 with 902 marker genes. (c) Top GO terms for the 881 APA features identified by spvAPA in SC-MOB1. BP, biological process; CC, cellular component; MF, molecular function. (d) Venn diagram showing the overlap of GO terms derived from the APA features identified by spvAPA and the collected 3088 important genes for the olfactory system in SC-MOB1. (e) UMAP visualization by integrating the gene expression and APA modalities using sPLS + UMAP and Seurat + UMAP, respectively.

Next, we proceeded with the single-cell mouse olfactory bulb (MOB) data (SC-MOB1 and SC-MOB2) to demonstrate the ability of spvAPA in selecting APA features distinguishing cell types in a supervised manner. Firstly, we utilized the imputation module of spvAPA to obtain the matrix ${\varnothing}^{+}$ for SC-MOB1 data. Then, we employed the feature selection module based on sPLS-DA in spvAPA to identify distinctive APA features of different cell types of MOB. In total, we obtained 11 components and 881 nonredundant APA features from SC-MOB1 (Supplementary Table 2). Each component could be considered as a meta-gene, and the overall score of each meta-gene is a linear combination of the APA features of the corresponding component. Then, cell type–specific APA features were identified in each meta-gene, according to the contributions of APA features in the loading matrix obtained by sPLS-DA (Supplementary Fig. 10). For example, the meta-gene corresponding to the first component (comp 1) scores lower in red blood cells (RBCs) than in other cell types; the meta-gene of the fourth component (comp 4) scores lower in endothelial cells (ECs) but higher in microglia (MicroG). Through UMAP visualization of genes with the highest contribution in each component, it can be observed that these genes exhibit differential usage preferences of APA sites in specific cell types (Fig. 6a). For instance, the RUD score ($\boldsymbol{\mathrm{\varphi}}$) for *SEMA3D* in RBCs is much higher than in other cell types, indicating a preference for using the distal poly(A) site in RBCs. *SEMA3D* has been reported to encode a member of the semaphorin III family, which was involved in axon guidance during neuron development [40].

Comparing the 881 APA features identified in SC-MOB1 with 902 marker genes for 12 cell types identified from the single-cell gene expression profile [23] (Supplementary Table 3), only 49 genes overlapped (Fig. 6b). The 881 APA features identified in SC-MOB1 were enriched in 1248 gene ontology (GO) terms (Fig. 6c and Supplementary Table 4), with the majority of these GO terms being closely related to synapse organization and neuron development. For example, the GO term with the lowest adjusted *P*-value (${\boldsymbol{P}}_{\mathbf{adj}}=\mathbf{2}\times{\mathbf{10}}^{-\mathbf{10}}$), GO:0016358, is related to dendritic development of neural cells and involves 39 APA genes. As a comparison, we also conducted GO enrichment analysis for the collected 3088 important genes of the olfactory system (Supplementary Table 3) and obtained a total of 3314 significantly enriched GO terms (Supplementary Fig. 11 and Supplementary Table 5). Of the 1248 GO terms derived from the APA features, 73.5% (917) were found among the pool of 3314 GO terms (Fig. 6d). Notably, there was substantial overlap between the top 50 GO terms enriched in both gene sets (APA features and collected genes), with 48 GO terms in common. In addition, the APA features were enriched in seven KEGG (Kyoto Encyclopedia of Genes and Genomes) pathways (Supplementary Table 6), among which five pathways were found in the pathways using the collected 3088 important genes (Supplementary Table 7). Particularly, the Polycomb repressive complex pathway enriched exclusively with APA features has been found crucial in regulating basal cell fate during adult olfactory neurogenesis [41]. These results suggest that APA features identified based on APA profiles constitute a distinct set of genes compared to the marker genes derived from traditional gene expression analysis, yet both contribute substantially to the primary functions of the olfactory system. Therefore, APA dynamics are independent of gene expression and represent an important source of cell–cell heterogeneity in the olfactory bulb.

Further, we found that the inclusion of APA features can improve the dimensionality reduction visualization of scRNA-seq data, and different modalities (i.e. matrix $\boldsymbol{G}$ and matrix ${\varnothing}^{+}$) may have varying or complementary contributions to the differentiation of different cell types. Detailed results are described in Supplementary Text Note 2 and Supplementary Fig. 12. Moreover, the semisupervised dimensionality reduction and visualization scheme of sPLS + UMAP provided in the spvAPA framework could effectively integrate multimodal data, facilitating dimensionality reduction and visualization. Detailed results are described in Supplementary Text Note 3, Fig. 6e, and Supplementary Fig. 12.

### spvAPA identifies spatially resolved alternative polyadenylation features from spatial transcriptomics data

**Figure 7:**
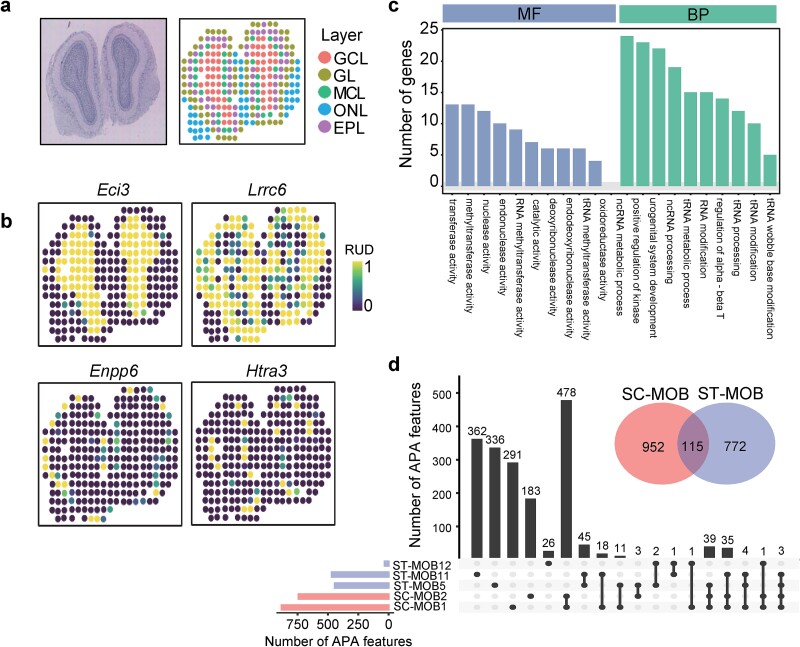
Analysis of spatial transcriptomics data with spvAPA. (a) Hematoxylin & eosin stain image and the layer annotation of the mouse olfactory bulb data ST-MOB11. (b) RUD scores for genes with the highest contribution in each component identified by the sPLS-DA module in spvAPA. (c) Top GO terms for the 468 APA features identified by spvAPA in ST-MOB11. BP, biological process; MF, molecular function. (d) Upset plot showing the overlap of APA features identified from the two replicates of SC-MOB and the three sections of ST-MOB. The bar chart shows the number of APA features from different combination of datasets. The black dots and lines represent the combination of datasets. The smaller bar chart on the left displays the total number of APA features from each dataset. The embedded Venn diagram shows the overlap between nonredundant APA features obtained from the two replicates of SC-MOB and those obtained from the three sections of ST-MOB.

Having demonstrated that spvAPA can effectively identify APA features from scRNA-seq data, next, we examined the applicability of svpAPA on spatial transcriptomics data using ST-MOB data as an example. The MOB is arranged in layers including the olfactory nerve layer (ONL), glomerular layer (GL), external plexiform layer (EPL), mitral cell layer (MCL), and granular cell layer (GCL) (Fig. 7a). First, we identified poly(A) sites from ST-MOB and obtained the APA usage matrix $\varnothing$. Then, we recovered missing entries in the matrix $\varnothing$ to obtain the matrix ${\varnothing}^{+}$ by the imputation module of spvAPA. The matrix ${\varnothing}^{+}$ was further processed by the sPLS-DA module in spvAPA to identify spatially resolved APA features distinguishing morphological layers (Supplementary Table 8). For ST-MOB11, four components were obtained, with the optimal number of APA features for each component being 300, 260, 50, and 18, respectively. Each component could be considered as a meta-gene, and the average RUD score of each meta-gene in each spot was then computed from the matrix ${\varnothing}^{+}$, denoted as meta-*φ*. Visualization of the meta-*φ* values for the four components demonstrated distinct spatial APA patterns (Supplementary Fig. 13). For example, the meta-*φ* value of the first component (comp 1) decreased from the outer to the inner layers; the second component (comp 2) showed lower meta-*φ* value in the ONL layer and higher in the EPL layer, with moderate level in other layers; the third component (comp 3) exhibited low meta-*φ* value in the GL layer and high in the EPL layer, with moderate in the remaining layers; and the fourth component (comp 4) showed higher meta-*φ* value exclusively in the MCL layer. We then selected the most contributive genes from each component (Fig. 7b). *Eci3* is a gene involved in lipid formation and the recycling of dopamine (DA) vesicles at synapses, as well as affecting synaptic nuclear protein metabolism [42]. The *φ* values of *Eci3* were close to 1 in the GCL and MCL layers and near 0 in other layers, indicating a preference for distal poly(A) site in the GCL and MCL layers. *Lrrc6* shows a preference for proximal poly(A) site in the ONL and GCL layers. *Htra3* is a gene involved in intracellular protein hydrolysis and a paralog of *Htra1* and was identified as a biomarker for Alzheimer’s disease in mice through proteomic analysis [43]. It prefers using the distal poly(A) site in the GL layer. *Enpp6*, involved in the myelination of neuronal axons [7], shows a preference for the distal poly(A) site in the MCL layer

To further select more robust APA features, we utilized the framework of 10-fold cross-validation using sPLS-DA (see Materials and Methods in Supplementary Text). A total of 468 genes were retained, with 258, 208, 35, and 12 APA features for the four components, respectively. These APA features enriched in 214 GO terms (Fig. 7c and Supplementary Table 9). Interestingly, most of these GO terms were related to transcription, which was different from the GO result of SC-MOB1 (Fig. 6c). A recent study by Navarro *et al.* [44] on ST-MOB conducted GO analysis for morphological layer-specific expression genes and found that a third of the biological process (BP) terms were related to transcription. This suggests that APA features that distinguish morphological layers of the olfactory bulb might predominantly affect activities such as transcription.

Next, we identified the APA features of the other two tissue sections of ST-MOB (ST-MOB5 and ST-MOB12). Surprisingly, the number of APA features recognized in ST-MOB12 (only 31) was much less than the other two sections (ST-MOB5 = 443; ST-MOB11 = 468) (Fig. 7d). However, it is indeed evident from the low-dimensional embeddings of the gene expression matrix and/or APA matrix that the separation of layers in ST-MOB12 is substantially lower than that in ST-MOB5 or ST-MOB11 (Fig. 4b and Supplementary Fig. 7), making it difficult to identify clear APA features for ST-MOB12. In contrast, 881 and 742 APA features were identified from SC-MOB1 and SC-MOB2, with 478 overlaps, which is much higher than the overlap of the three sections in ST-MOB (Fig. 7d). This indicates that the difference among different sections of spatial transcriptome may be much greater than that among different replicates of scRNA-seq data. Moreover, a total of 1067 nonredundant APA features were obtained from SC-MOB, with only 115 overlaps with APA features identified from ST-MOB (Fig. 7d). Such a low overlap rate suggests that only ~10% of APA genes identified based on prior information in the morphological layer may be explained by cell type specificity, and the vast majority of the identified APA features are due to spatial differences rather than differences in cell type.

## Discussion

Poly(A) sites are associated with genes, and the modality of APA and gene expression are not independent. In our pipeline, we employed the RUD matrix $\varnothing$ instead of the poly(A) site expression matrix to represent the APA modality. This is because, for genes with only one poly(A) site, their poly(A) site expression approximates the gene-level expression, which would introduce redundant information when using the poly(A) site expression matrix. In contrast, the matrix $\varnothing$ represents variations of the 3′ UTR length across cells/spots, providing complementary information to the gene expression profile. However, the matrix $\varnothing$ is even sparser than the poly(A) site or gene expression matrix, therefore, utilizing imputation methods to restore authentic APA dynamics holds significant importance. The evaluation results demonstrated the superiority of the WNN-based imputation module provided in spvAPA (Figs 2 and 3, and Supplementary Fig. 1). Existing tools for imputing the APA matrix ∅ used only a single modality to identify nearest neighbor cells; stAPAminer used only the matrix *G,* and scDaPars used only the matrix ∅. However, the information from a single modality is limited. For instance, in some heterogeneous cells, gene expression profiles may be similar but APA profiles may differ greatly. In such case, relying solely on gene expression profiles (the matrix *G*) to determine nearest neighbor cells will lead to erroneous estimation of cell–cell similarity. Previous studies have revealed hidden cell subpopulations invisible to gene expression [13, 15, 45, 46]. Our analysis on the SC-MOB data revealed that the gene expression profile and APA profile each captured distinct sets of cell-type-specific genes (Figs. 6b–d and Supplementary Fig. 12). Different from scDaPars or stAPAminer, spvAPA integrated both gene expression modality and APA modality to extract cell type heterogeneity from different aspects, thereby accurately identifying nearest neighbor cells for recovering missing APA signatures.

Many existing bioinformatic methods for analyzing single-cell and spatial transcriptomics data focus only on gene level, such as detecting DEGs or SVGs based on gene expression. There are also a few emerging studies that identify DEAPA genes among different cell types or specific to spatial domains, but almost all use nonparametric statistical testing methods such as the Wilcoxon rank-sum test or the chi-square test [12, 14, 45–47]. Although nonparametric tests may be efficient in some cases, they consider only individual genes and ignores potential relationship between genes. Moreover, due to the fact that the APA profile is represented as ratios rather than counts, and many gene-level tools used for DEG or SVG detection require data to follow a certain distribution such as Poisson or negative binomial distribution [18, 48], they are not suitable for APA analysis. The sPLS-DA module implemented in spvAPA is a multivariate prediction method that uses prior category labels for APA feature selection, which can simultaneously consider multiple genes and solve the multicollinearity problem in high-dimensional data without requiring any data distribution assumptions. Therefore, spvAPA is highly suitable for identifying APA markers from single-cell and spatial transcriptomics data that are extremely sparse and noisy (Fig. 6). It should be noted that the proposed spvAPA is a supervised method that can identify and highlight differences between specified categories, which is not applicable to experiments without prior labels. However, due to the fact that many single-cell or spatial datasets contain rich metadata information (e.g. cell type information, developmental time, and cell cycle), spvAPA has broad applicability. For data lacking metadata information, various automatic cell annotation methods [49] can be used to infer cell types through the utilization of curated marker gene databases [50], the correlation with reference expression data [51], or the application of supervised classification for label transfer [52]. It should be also noted that, as a linear method, PLS-DA cannot capture nonlinear relationships, potentially limiting its application in certain datasets. However, despite the importance of nonlinear methods in computational biology, linear methods can have comparable performance to machine learning approaches, as highlighted in previous research [53]. Furthermore, studies have shown that simple, linear models often perform satisfactorily, even for nonlinear data [21, 54–56]. Moreover, similar to the principal components (PCs) obtained from PCA (principal component analysis), PCs derived from PLS-DA can also be utilized in the processing and analysis of single-cell data, which can be fed into methods like UMAP to produce nonlinear embeddings.

Generally, the visualization module of spvAPA is highly flexible, which could be executed in an unsupervised, semisupervised, or supervised manner. The recovered APA profile from spvAPA can be used solely for visualization through unsupervised methods like UMAP (Fig. 4 and Supplementary Fig. 7). The visualization module can also use genes or APA features identified by supervised framework—PLS-DA or sPLS-DA. It also includes a semisupervised dimensionality reduction and visualization strategy combining sPLS-DA and UMAP, which can integrate the gene expression modality and APA modality for enhanced visualization. Most dimensionality reduction algorithms, such as UMAP and PCA, are unsupervised and ignore existing label information. Using spvAPA, even in a single dataset, different cell labels can be used to visualize the same cells in multiple graphs. As such, each graph can highlight the cellular heterogeneity associated with a specific biomarker. Therefore, spvAPA can benefit from information related to experimental design and sample metadata, utilizing the metadata to gain new biological insights. However, admittedly, given the diversity of techniques used in single-cell or spatial transcriptomics, there is no single method that drives optimal visualization and interpretability for all data, and different methods may have varying performance rankings using different metrics [20]. It is encouraged to incorporate PCA, UMAP, and other algorithms to benefit from the unique advantages of each algorithm. Although only the results of the sPLS-DA + UMAP stage were presented in this study, other combinations such as PLS-DA + UMAP, PCA + PLS-DA, and PCA + PLS-DA + UMAP could also be easily implemented and examined. In addition, when simplifying high-dimensional data into two dimensions, information is always lost. Therefore, visualization is only one aspect of data exploration analysis, which should be accompanied by further quantitative and high dimensional analysis.

The single-cell datasets used in this study were all from scRNA-seq rather than single-nucleus RNA-seq (snRNA-seq). Previous studies based on gene-level analysis have revealed that, comparable to scRNA-seq, snRNA-seq generally performed well for sensitivity and classification of cell types [57, 58]. However, to the best of our knowledge, there is currently no research comparing the differences between snRNA-seq and scRNA-seq in APA profiling. In fact, there are currently very few studies specifically using snRNA-seq data for APA analysis. Previously, Agarwal *et al.* used a dataset comprising single nucleus transcriptional profiling of ~2 million nuclei of mouse embryonic development [59] and revealed the landscape of APA in single cells of the developing mouse embryo. Wang *et al.* investigated dynamic APA usage in different cell types using the snRNA-seq data of 1424 human brain cells [60]. These studies demonstrate that snRNA-seq data can be used to extract APA sites and analyze APA dynamics across cell types or developmental stages. In principle, as long as APA sites can be extracted, spvAPA can be used for subsequent applications such as APA signature imputation, APA feature selection, and integrative visualization. Nevertheless, it would be interesting to conduct another comparative study in the future to compare the effectiveness of spvAPA on scRNA seq and snRNA seq data.

In addition to APA, alternative splicing (AS) of pre-mRNAs also greatly contributes to transcriptome diversity. The coordination and competition between AS and APA have been reported [61–63]. The percent spliced in (PSI) index that represents the ratio between reads including or excluding exons was commonly used in computational methods for AS [64]. Similar to the APA ratio, the PSI index is also a ratio value between 0 and 1. We speculate that spvAPA is also applicable for recovering missing entries in PSI data and can be used to integrate PSI with gene expression data for downstream analysis and visualization. In the future, additional work will be conducted using more data from different species to test the applicability of spvAPA to AS data. Particularly, it should be noted that the degree of compositionality varies across modalities of gene expression, APA, and AS because they represent proportions or relative values constrained by fixed totals. The current version of spvAPA does not fully address the compositional nature of gene expression and APA data, and it remains unclear how compositionality impacts the results in this study. In the future, specialized analysis techniques [65–68] could be incorporated for compositional single-cell data integration to resolve compositional constraints and extract meaningful biological insights. Moreover, how to integrate compositional data from more modalities such as AS, APA, and gene expression for more accurate cell identity determination is also a direction worth exploring.

**Key Points**

- spvAPA is the first tool to explore alternative polyadenylation (APA) in a supervised manner from single-cell and spatial transcriptomics data.
- spvAPA integrates complementary information from modalities of APA or gene expression, performing better than methods relying on a single modality.
- spvAPA is capable of recovering missing APA signatures, identifying APA features and marker genes, facilitating interpretable visualization, and discovering novel cell subtypes.
- Evaluation using nine single-cell and spatial transcriptomics datasets demonstrates the effectiveness and applicability of spvAPA.

## Supplementary Material

Supplementary_Figures_bbae720

Supplementary_Tables_bbae720

Supplementary_Text_bbae720

## Data Availability

The datasets supporting the results of this article are available in https://github.com/BMILAB/spvAPA.
