# Supplementary material for: Supervised analysis of alternative polyadenylation from single-cell and spatial transcriptomics data with spvAPA
Source: Brief Bioinform. 2025 Jan 11;26(1):bbae720. doi: 10.1093/bib/bbae720 (PMC11724721; doi:10.1093/bib/bbae720)
Supplement: Supplementary_Figures_bbae720 [file supplementary_figures_bbae720.pdf]

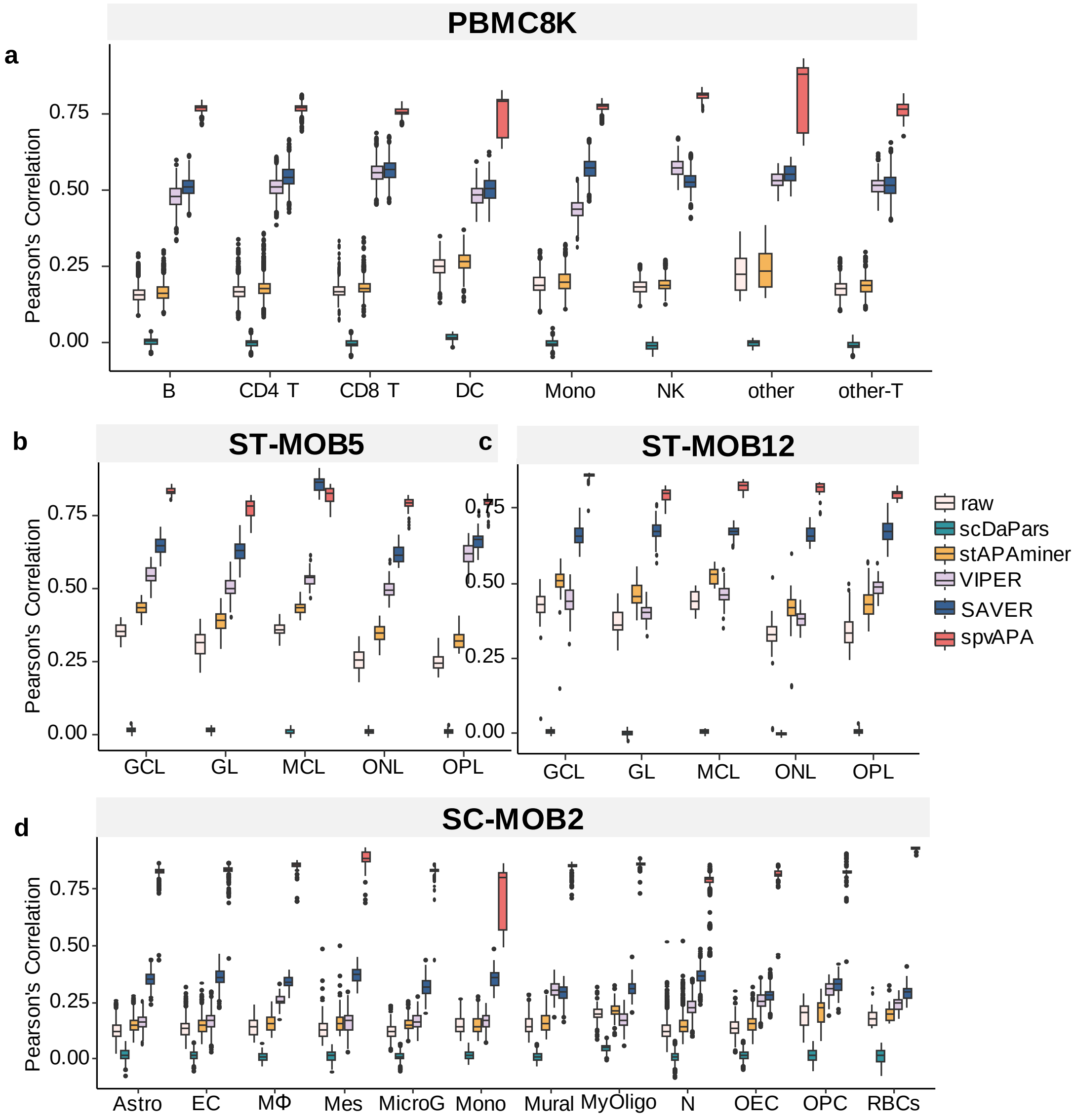

Supplementary Figure 1. Pearson's correlations estimated using imputed $\emptyset^{+}$ matrices by different methods and the raw $\emptyset$ matrix of PBMC8K (a), ST-MOB5 (b), ST-MOB12 (c), and SC-MOB2 (d). For each dataset, the Pearson's correlation of the APA profile of each cell/spot in each cell type/layer with the average APA profile of the raw data in the respective cell type/layer were calculated.

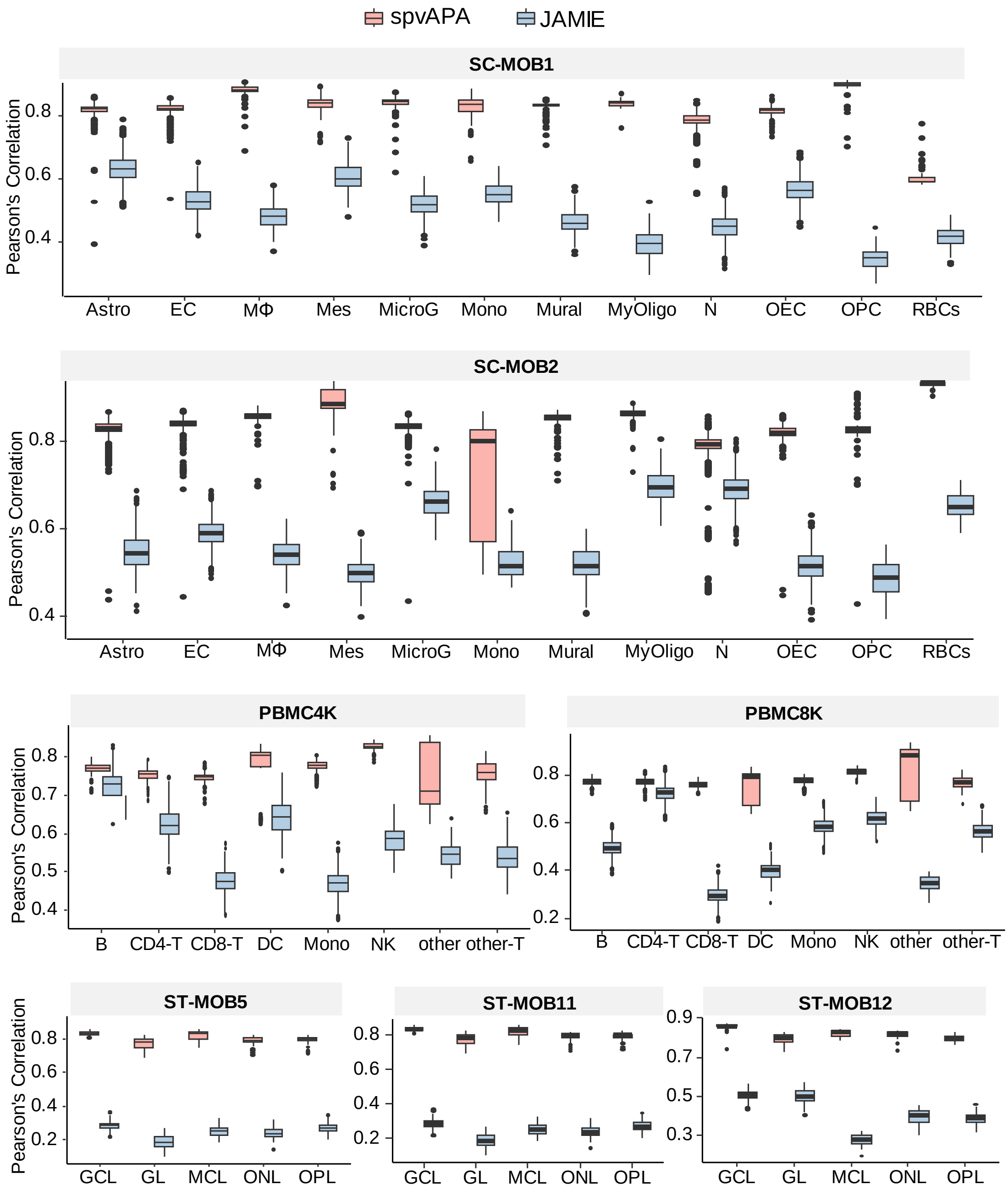

Supplementary Figure 2. Pearson's correlations estimated using the WNN module in spvAPA and the JAMIE tool. For each dataset, the Pearson's correlation of the APA profile of each cell/spot in each cell type/layer with the average APA profile of the raw data in the respective cell type/layer were calculated. Details are provided in Supplementary Text Note 1.

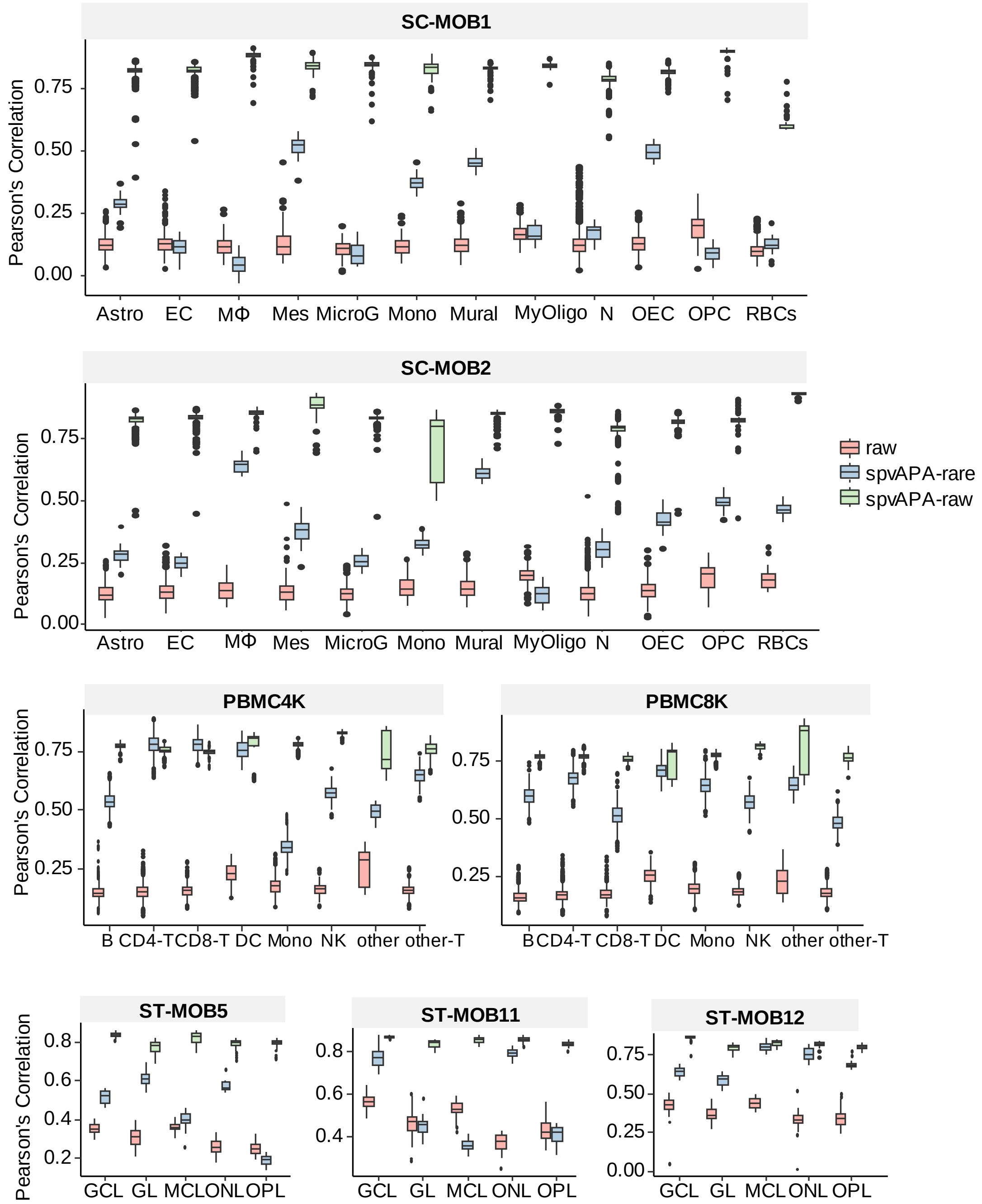

Supplementary Figure 3. Pearson's correlations estimated using the WNN module on imbalanced data. "Raw" means the correlations calculated using the raw data without imputation. "spvAPA-rare" means the correlations calculated using the imbalanced data after imputation with spvAPA. "spvAPA-raw" means the correlations calculated using the raw data after imputation with spvAPA. Details are provided in Supplementary Text Note 1.

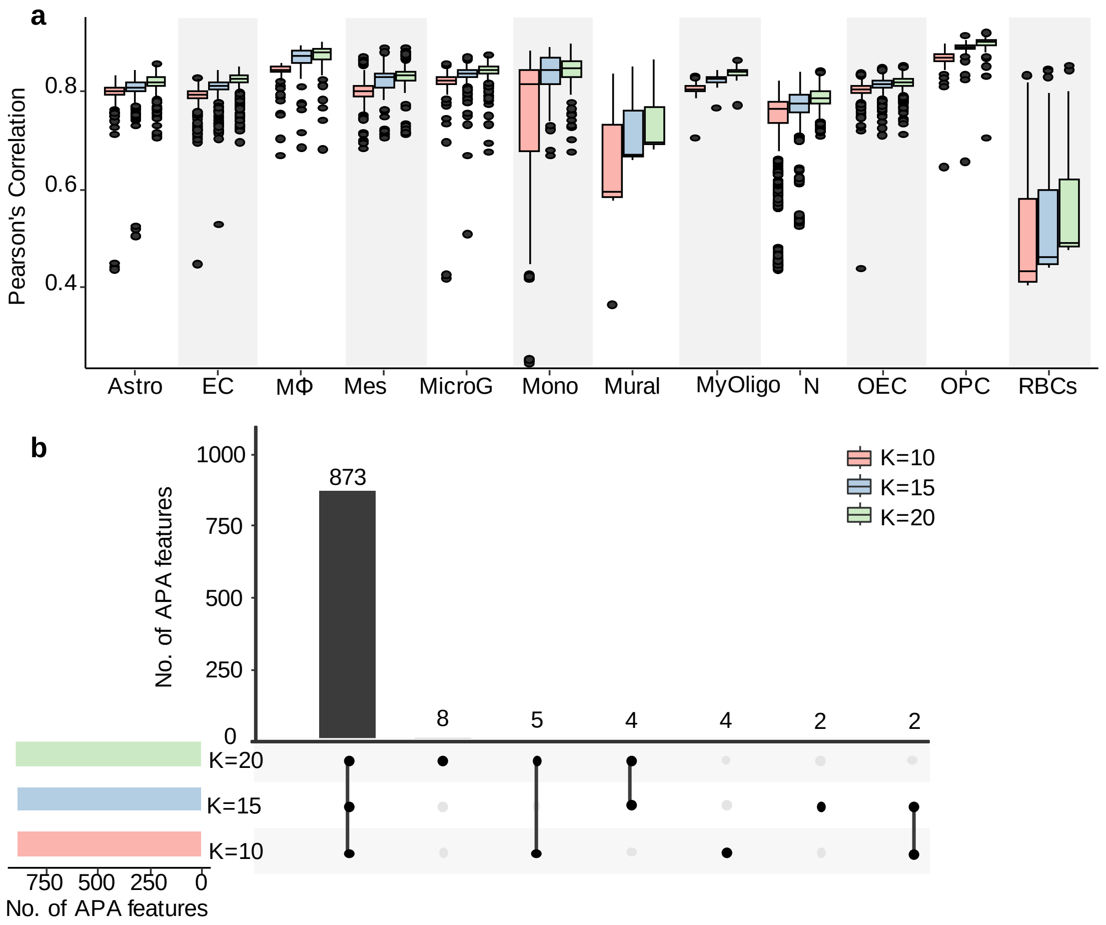

Supplementary Figure 4. Evaluation of the WNN module with different k values. **a** PCC values calculated based on APA matrices imputed using the WNN module with different k values. **b** APA features selected by sPLS-DA on APA matrices imputed using the WNN module with different k values. Details are provided in Supplementary Text Note 1.

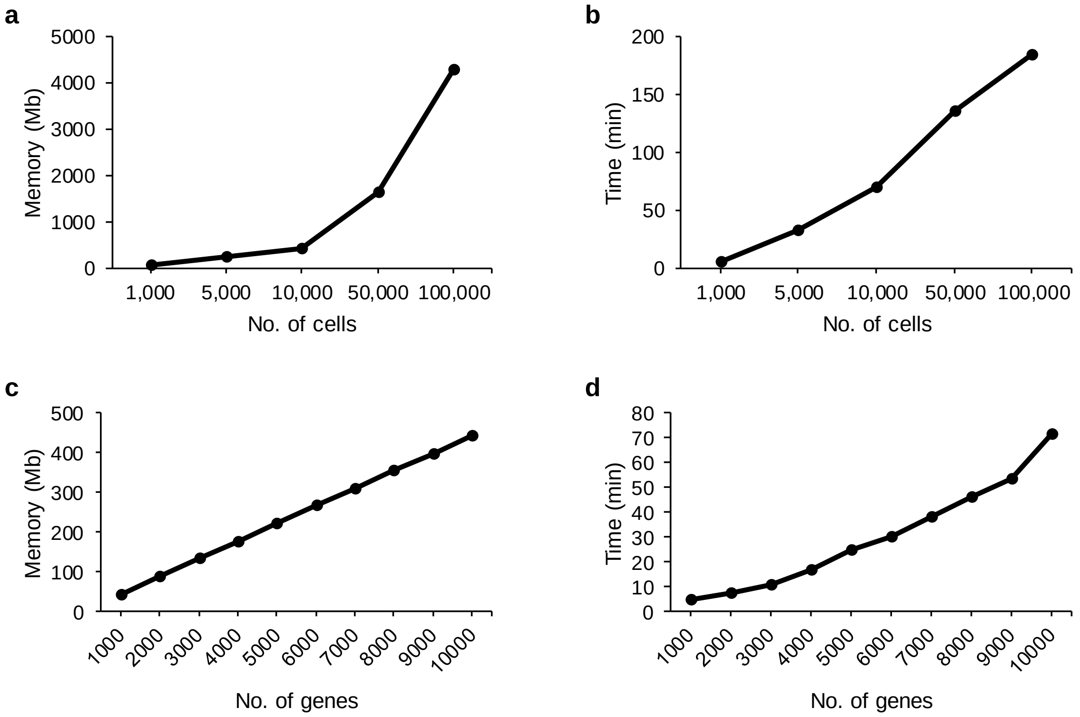

Supplementary Figure 5. Evaluation of computational efficiency of the WNN-based imputation module of spvAPA on datasets of different sizes. a Memory usage on datasets containing 5000 genes and varying number of cells from 1000 to 100,000. **b** Run time on datasets containing 5000 genes and varying number of cells from 1000 to 100,000. **c** Memory usage on datasets containing 5000 cells and varying number of genes from 1000 to 10,000. **d** Run time on datasets containing 5000 cells and varying number of genes from 1000 to 10,000.

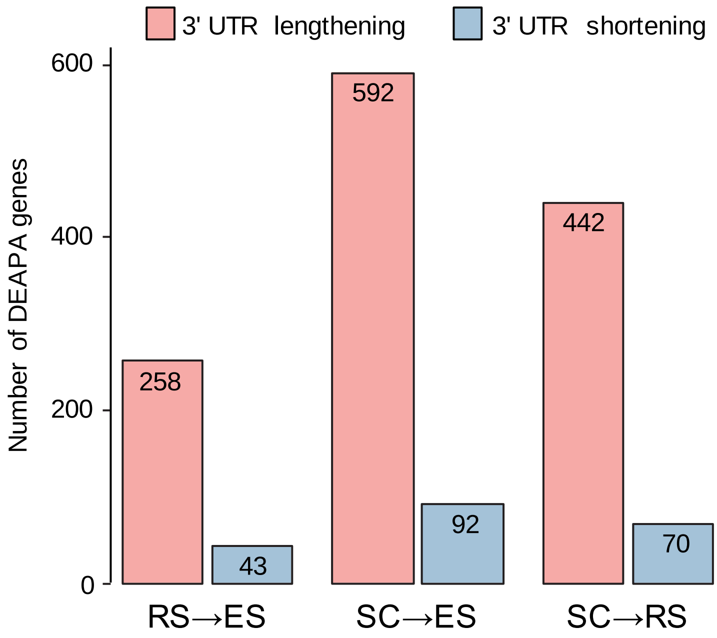

Supplementary Figure 6. Number of genes with differentially used APA sites (DEAPA genes) among three developmental states, spermatocytes (SC), round spermatids (RS), and elongating spermatids (ES) during mouse spermatogenesis. The two bars of "RS→ES" mean that there are 258 DEAPA genes with significant 3' UTR lengthening from RS to ES, and 43 DEAPA genes with significant 3' UTR shortening from RS to ES, respectively.

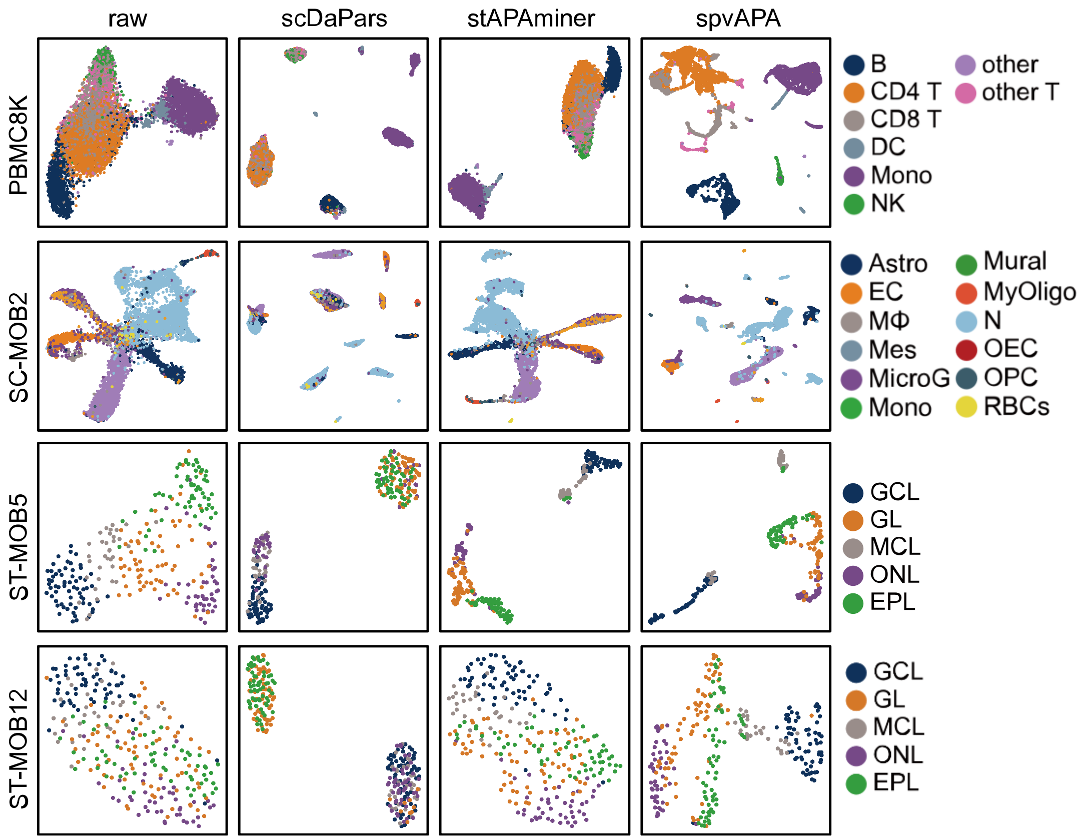

Supplementary Figure 7. UMAP visualization for the raw $\emptyset$ matrix or imputed $\emptyset^{+}$ matrices by different methods for PBMC8K, SC-MOB2, ST-MOB5, and ST-MOB12. For each matrix, normalization and dimensionality reduction with principal component analysis were first performed by Seurat before UMAP visualization.

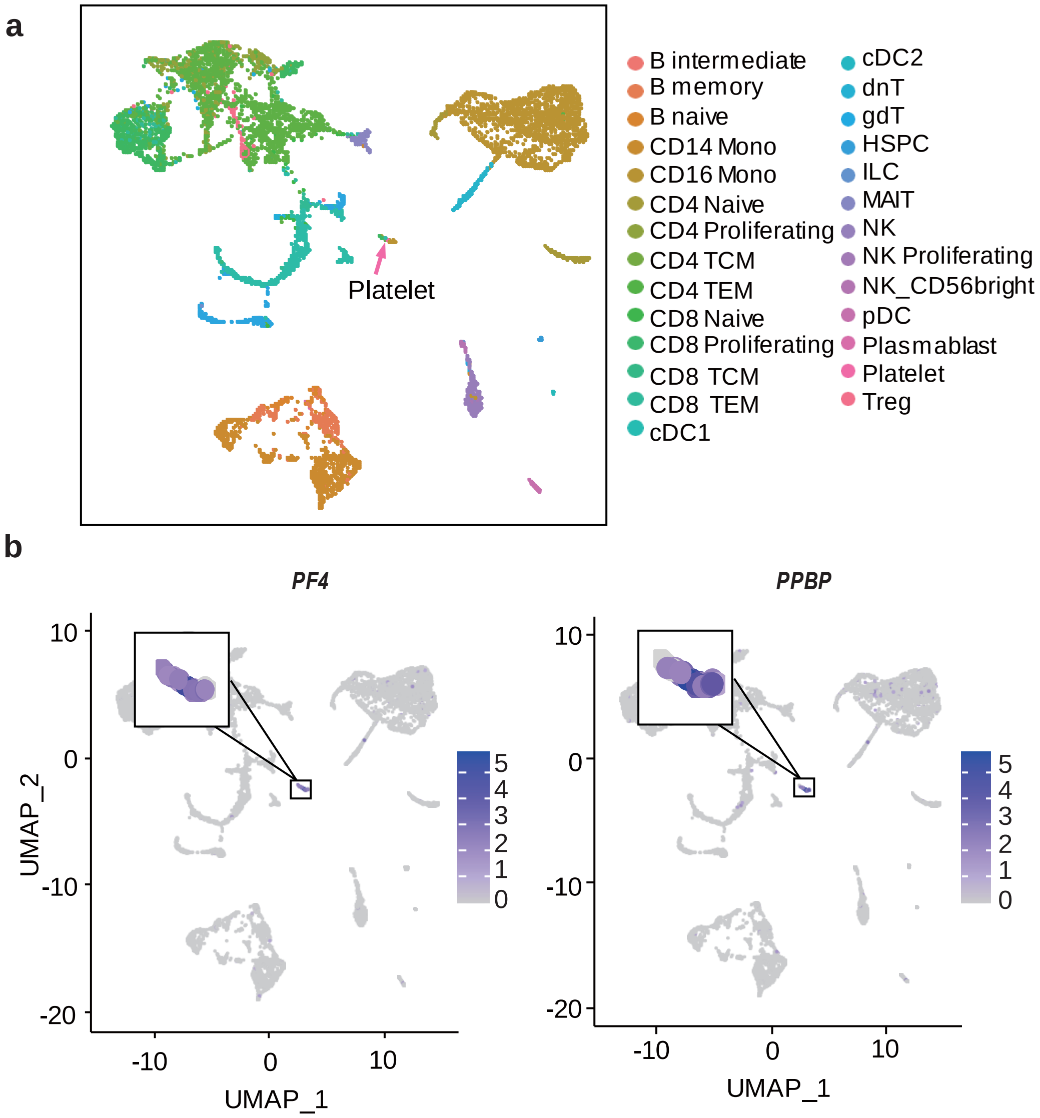

Supplementary Figure 8. Discovery of sub-cell types from PBMC8K with spvAPA. **a** UMAP plot showing a small cluster of platelets in PBMC8K. The arrow mark the cluster of platelets. **b** UMAP plot showing the expression of two platelet-specific marker genes, *PF4* and *PPBP*. The box marks the cluster of platelets

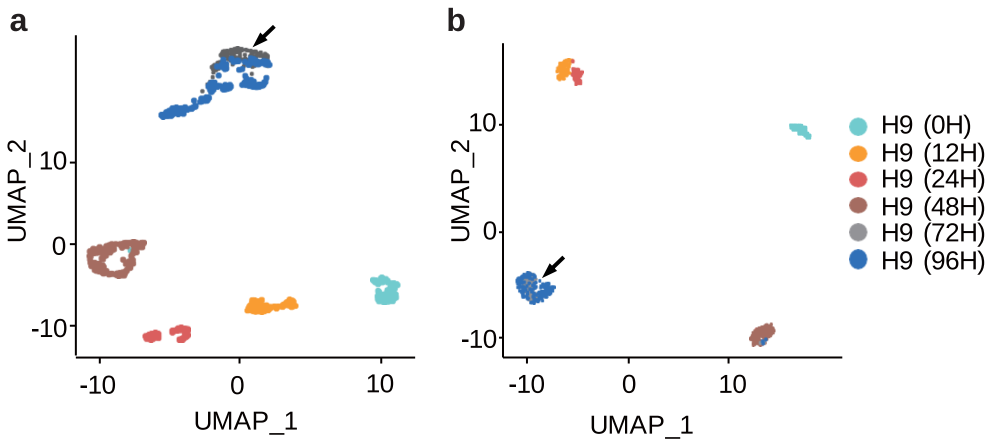

Supplementary Figure 9. UMAP visualization of the hESC dataset. a UMAP plot using the matrix $\emptyset^{+}$ generated by spvAPA clearly separates human embryonic stem cells of different differentiation times. **b** UMAP plot using the matrix $\emptyset^{+}$ generated by scDaPars failed to separate human embryonic stem cells differentiated for 72 and 96 hours. The arrow marks the clusters of 72 and 96 hours

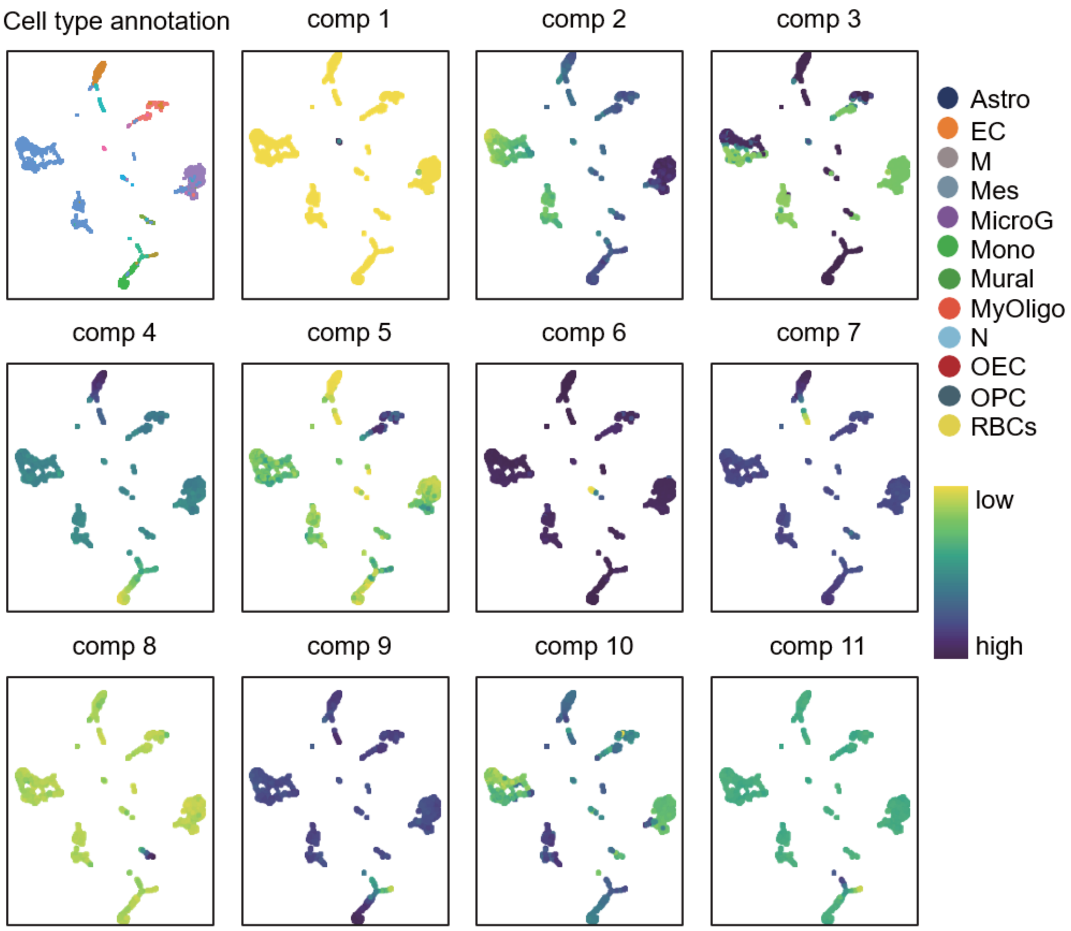

Supplementary Figure 10. UMAP plots for the single-cell MOB data showing the component loadings for each component determined by the sPLS-DA module in spvAPA. The first UMAP plot shows the cell type annotation. Each component could be considered as a meta-gene, and the overall score of each meta-gene is a linear combination of the APA features of the corresponding component.

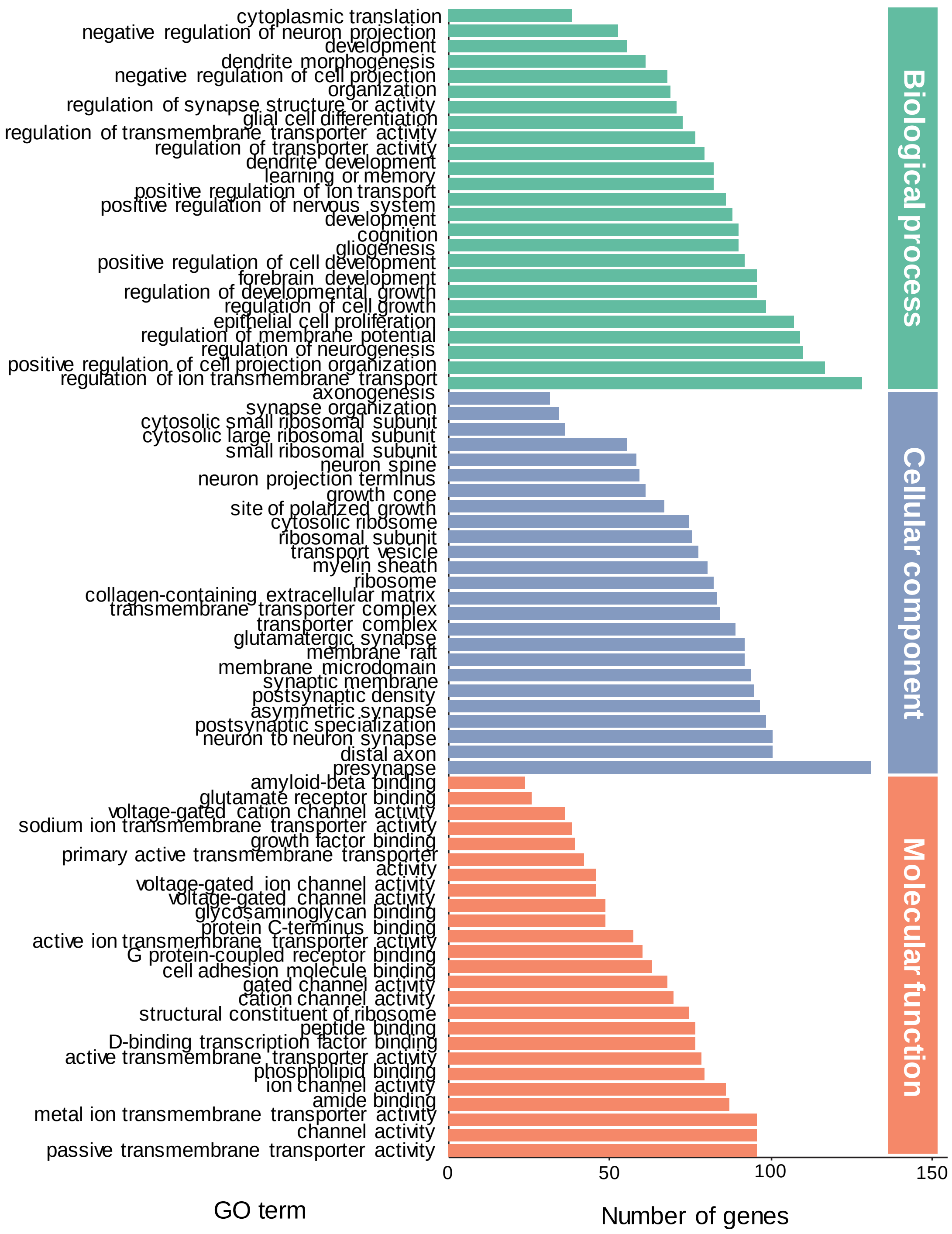

Supplementary Figure 11. Top GO terms obtained using the collected 3088 important genes for the olfactory system bulb.

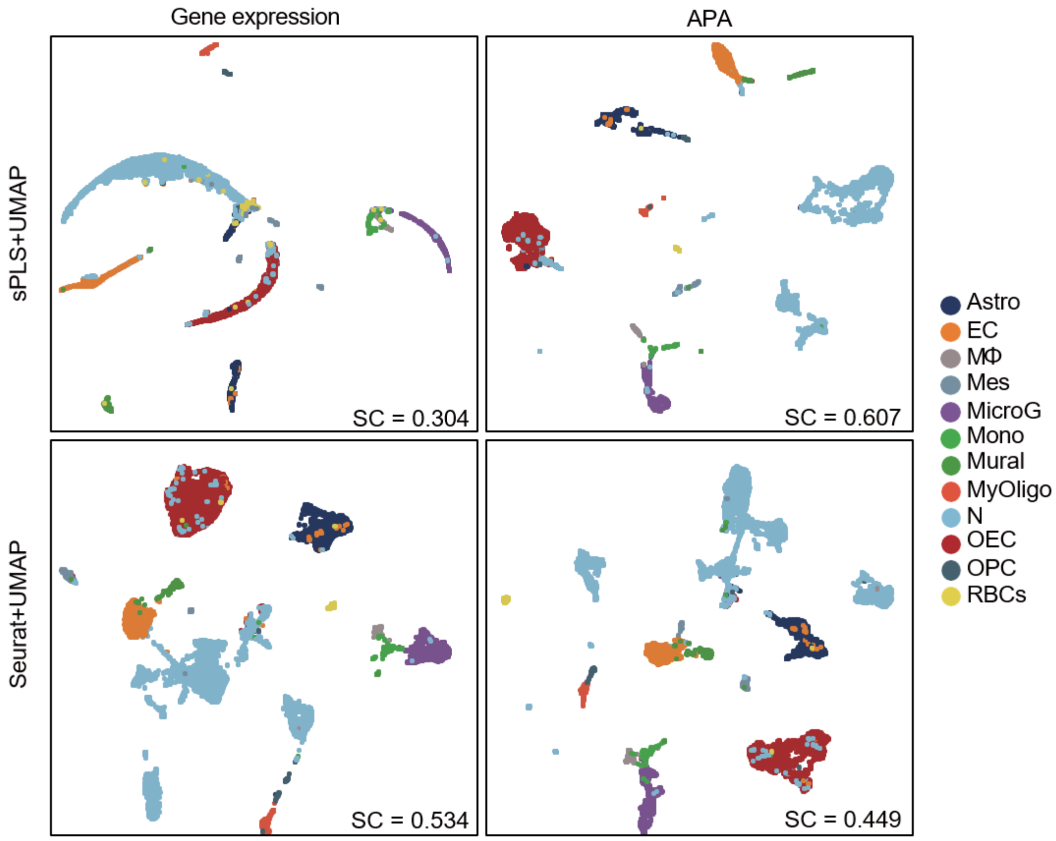

Supplementary Figure 12. UMAP visualization for the single-cell MOB data using the gene expression modality or the APA modality with sPLS+UMAP or Seurat+UMAP. sPLS+UMAP means that the low dimensional embeddings obtained by sPLS-DA module in spvAPA were used as the input for UMAP visualization. Seurat+UMAP means that the low dimensional embeddings obtained by Seurat were used as the input for UMAP visualization. The "gene expression" column displays the plot using the gene expression only, while the "APA" column displays the plot using the spvAPA-imputed APA matrix. The corresponding silhouette coefficient (SC) score was shown on each plot.

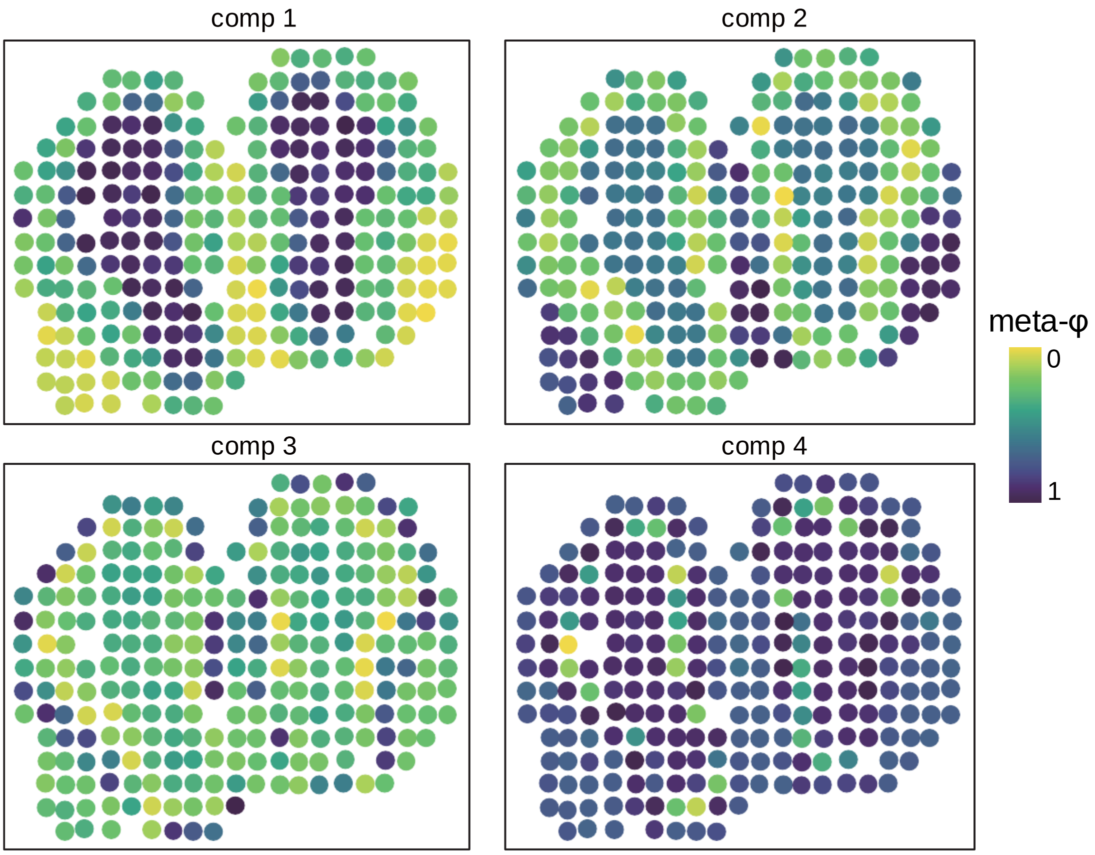

Supplementary Figure 13. UMAP plots showing the component loadings for each component determined by the sPLS-DA module in spvAPA for ST-MOB11 data. Each component could be considered as a meta-gene, and the average RUD score of each meta-gene in each spot was then computed from the matrix $\emptyset^{+}$, denoted as meta-φ. The meta-φ ranges from 0 to 1.
