# Supplementary material for: Supervised analysis of alternative polyadenylation from single-cell and spatial transcriptomics data with spvAPA
Source: Brief Bioinform. 2025 Jan 11;26(1):bbae720. doi: 10.1093/bib/bbae720 (PMC11724721; doi:10.1093/bib/bbae720)
Supplement: Supplementary_Text_bbae720 [file supplementary_text_bbae720.pdf]

Supervised analysis of alternative polyadenylation from single-cell and spatial transcriptomics data with spvAPA

Supplementary Text

Materials and Methods 2

Data 2

Computing the matrix $\emptyset$ from single-cell and spatial transcriptomics data 3

Imputation of missing entries in the APA matrix $\emptyset$ 4

Supervised identification of APA features 6

Dimensionality reduction, clustering, and visualization by integrating multi-modal data 9

Evaluation Metrics 11

Note 1: evaluating the WNN-based imputation module in spvAPA 11

Note 2: using APA features to improve the dimensionality reduction visualization of scMOB data 14

Note 3: integrating APA and gene modalities to enhance dimensionality reduction and visualization 15

References 17

Materials and Methods

Data

A total of nine single-cell and spatial transcriptomics datasets were analyzed in this study (Supplementary Table 1). The scRNA-seq data include two datasets of mouse olfactory bulb (SC-MOB1 and SC-MOB2) [1], a mouse spermatogenesis dataset [2], a hESC dataset [3], and two datasets of human peripheral blood mononuclear cells (PBMC4K and PBMC8K). Cell type annotations for SC-MOB were obtained from the original study [1]. Raw sequencing data of SC-MOB and mouse spermatogenesis were processed using CellRanger. Sequence alignment results in BAM (Binary Alignment Map) format and gene expression matrices for PBMC4K and PBMC8K were obtained from 10x Genomics (https://www.10xgenomics.com/), with cell types identified via label transfer using Azimuth [4] (https://azimuth.hubmapconsortium.org/). The hESC dataset was obtained by Smart-Seq2 sequencing. The APA $\emptyset$ matrix was sourced from the study of scDaPars [5] (https://github.com/YiPeng-Gao/scDaPars), and the gene-cell expression matrix and cell type annotations were retrieved from NCBI GEO (National Center for Biotechnology Information Gene Expression Omnibus) under accession number GSE75748. The spatial transcriptomics data (ST-MOB) were obtained from the MOB tissue [6]. Three tissue sections of ST-MOB analyzed in previous studies [7-12] were used (rep5, rep11, and rep12), denoted as ST-MOB5, ST-MOB11, and ST-MOB12 (Supplementary Table 1). The morphological layer labels for ST-MOB were obtained through manual annotation, by

using the ST Spot Detector[13] to match spots with the H&E (Hematoxylin and Eosin) stained image. The gene-cell expression matrix, APA $\emptyset$ matrix, spatial location information, and morphological layer labels were obtained from the study of stAPAminer [7]. Additionally, we collected 3,088 marker genes associated with the MOB system (Supplementary Table 3), including 902 marker genes obtained from single-cell analysis representing 12 cell types [1], 10 experimentally validated mitral cell marker genes obtained from spatial transcriptomics data [6], and 2,326 genes for the olfactory system from the Harmonizome database [14].

Computing the matrix $\emptyset$ from single-cell and spatial transcriptomics data

For sequencing strategies targeting 3′ ends of the transcripts (*e.g.*, 10x Chromium [15] for scRNA-esq and 10x Visium [16] for spatial transcriptomics), scAPAtrap [17] was used for genome-wide identification and quantification of poly(A) sites. Of note, the spvAPA framework is flexible and can accommodate other existing tools instead of scAPAtrap, such as Sierra [18]. After poly(A) site identification and quantification, a poly(A) site expression matrix can be obtained, with each row denoting a poly(A) site and each column a cell/spot. We discarded poly(A) sites expressed in fewer than three cells/spots and cells/spots expressing fewer than 200 poly(A) sites. Then, movAPA [19] was adopted to annotate poly(A) sites with gene and genomic region information. As in the previous study [17], annotated 3′ UTRs were extended by 1000 bp to include more authentic poly(A) sites located immediately downstream of annotated 3′ UTRs. To mitigate the influence of

internal priming, we excluded poly(A) sites surrounding by six consecutive adenines or a total of seven adenines within a 10 nt window centering the site. Finally, we retained genes with at least two poly(A) sites in the 3′ UTR. Statistics of APA sites and APA genes for each dataset analyzed in this study were provided in Supplementary Table 10. The relative usage of distal poly(A) site (RUD) for 3′ UTR-APA gene *i* in cell/spot *s* was calculated as the ratio of the expression of the distal poly(A) site d to the sum of the expression of all poly(A) sites located in the 3′ UTR (Eq. 1).

$$RUD_{s,i} = \frac{E_{d,i}}{\sum E_i} \tag{1}$$

The RUD value ranges between 0 to 1, where a higher RUD value indicates an increased usage of distal poly(A) site in the gene. A higher RUD of a gene also indicates that the gene tends to utilize transcripts with longer 3′ UTR. Finally, an RUD matrix $\emptyset$ was obtained, where each row represents a 3′ UTR-APA gene and each column represents a cell/spot.

Imputation of missing entries in the APA matrix $\emptyset$

Due to the high dropout rates in scRNA-seq data and the scarcity of APA transcripts, the RUD matrix $\emptyset$ is even more sparse than the already sparse gene-cell expression matrix. To alleviate the computational challenges due to the high sparsity, we proposed a method based on weighted nearest neighbor (WNN) [4] to impute missing entries in the RUD matrix $\emptyset$. The rationality of using WNN method for imputation from multi-modal data is based on the assumption that similar cells have similar APA profile, and more accurate cell similarity can be inferred using more complementary information from multiple modalities.

Briefly, we integrated both gene expression modality and APA modality to identify neighboring cells/spots, and then used the mean value of similar cells/spots to impute the matrix $\emptyset$. The WNN-based imputation method consists of three steps: (1) constructing independent K-nearest neighbor (KNN) graphs for the gene expression modality and APA modality; (2) merging two modalities' KNN graphs to obtain a WNN graph using weighted nearest neighbor analysis; (3) inferring the nearest neighbor cells based on the WNN graph and employing the mean value of nearest neighbor cells to interpolate missing values in the matrix $\emptyset$. Notably, we employed the matrix $\emptyset$ instead of the poly(A) site expression matrix to represent the APA modality. This is because, for genes with only one poly(A) site, their poly(A) site expression approximates the gene-level expression, which would introduce redundant information when using the poly(A) site expression matrix. In contrast, the matrix $\emptyset$ represents variations of the $3'$ UTR length across cells/spots, providing complementary information to the gene expression profile.

The input of the WNN-based method is the gene-cell expression matrix G and the RUD matrix $\emptyset$. For matrix G, we used *SCTransform* function in the Seurat package [20] for normalization. Then we performed PCA to obtain the top principal components (PCs), then cell-cell similarities were calculated and a KNN graph was constructed. For the matrix $\emptyset$, we initially identified highly variable genes (HVGs) using the *vst* method in Seurat's *FindVariableFeatures* function, then scaled the data with Seurat's *ScaleData*. Then we applied PCA for dimensionality reduction and constructed a KNN graph based on cell-cell similarities calculated from the top PCs. Subsequently, we merged the two KNNs based on

the weighted nearest neighbor analysis to create a comprehensive WNN graph. Upon obtaining the WNN graph, an iterative process was employed to interpolate missing entries in matrix $\emptyset$.

Given an RUD matrix $\emptyset$ with m genes and n cells/spots, for each cell/spot, nearest k cells/spots (default $k = 20$) were recorded according to the WNN graph. In each iteration, for a gene with missing RUD value in matrix $\emptyset$, the average RUD value of this gene in these k cells/spots is calculated for imputation (Eq. 2). Of note, only spots with a non-zero RUD score for the gene were counted.

$$\varphi_{ij}^{+} = \frac{1}{L}\sum_{x\in[k]}\varphi_{ix} \text{ when } \varphi_{ij} \text{ is missing} \quad (2)$$

$$(i = 1, 2, \ldots, m;\ \ j = 1, 2, \ldots, n)$$

Here, φ_{ij}^{+} is the imputed RUD score for gene i in cell/spot j; $[k]$ denotes the set of nearest k cells/spots with an RUD score for the respective gene; L is the number of cells/spots in $[k]$ with non-zero RUD score in cell/spot j.

This iterative process continues until all missing values in the matrix $\emptyset$ were filled, or the maximum number of iterations (default is 10) was reached. Upon completion of the iteration process, any remaining missing entries in the matrix $\emptyset$ will be set to zero. The matrix $\emptyset$ after imputation is denoted as $\emptyset^{+}$.

Supervised identification of APA features

The number of samples (cells/spots) in single-cell and spatial transcriptomics data is often much smaller than the number of variables (genes), leading to potential severe

multicollinearity among independent variables. Moreover, meta data, such as cell type annotations and morphological layers, are often available in biological experiments, which could be included in data analysis to improve the interpretability and accuracy. Within the spvAPA framework, we referred to the PLS regression model for supervised selection of APA features from the matrix $\emptyset^{+}$. We implemented sparse PLS (sPLS) for variable selection and feature extraction by introducing $L1$ regularization term through Lasso (Least Absolute Shrinkage and Selection Operator). To apply prior labels for precise identification of pattern recognition corresponding to the prior categories, we further utilized sPLS discriminant analysis (sPLS-DA) which was implemented using the R package mixOmics [21].

Specifically, for the predictor variables $X_{n\times p}$ (where n represents the number of cells or spots, p represents the number of genes) and the continuous response variable $Y_{n\times q}$ (where q represents the number of categories), it was assumed in PLS analysis that both X and Y have latent variables $T_{n\times K}$ and $U_{n\times K}$, thus X and Y can be decomposed (Eq. 3).

$$\begin{aligned} \mathrm{X} &= \mathrm{TP}^{\mathrm{T}} + \mathrm{E} \\ \mathrm{Y} &= \mathrm{UQ}^{\mathrm{T}} + \mathrm{F} \end{aligned} \qquad (3)$$

Here, $P_{p\times K}$ and $Q_{q\times K}$ (where K represents the number of latent variables) are the loading matrices for X and Y respectively, indicating the contribution of variables to the predictive components. $E_{n\times p}$ and $F_{n\times q}$ are residual matrices, and T is the matrix of latent variables to be predicted, defined as $T = XW$, where $W_{p\times K}$ is the matrix of K direction vectors ($1 \leq K \leq min\{n, p\}$).

By introducing $L1$ regularization term, sPLS solves the following optimization problem

for the kth direction vector (Eq. 4).

$$\min_{w,\ c} -\kappa w^T M w + (1-\kappa)(c-w)^T M (c-w) + \lambda_1 \|c\|_1 + \lambda_2 \|c\|_2 \tag{4}$$

subject to $w^T w = 1$, where $M = X^T Y Y^T X$. This formulation applies the $L1$ penalty to the direction vector c instead of the original direction vector w.

Furthermore, we recoded known biological labels into a continuous dummy response matrix to implement a sparse version of sPLS-DA. Prior labels were recoded into the response matrix $Y_{n\times q}$ using dummy variables to represent the prior categories of each cell/spot. In this framework, we performed variable selection only on the predictor variables and the response matrix remains unchanged, *i.e.*, we aimed to select feature genes that help predict the categories of cells/spots. In sPLS-DA [22], two parameters require tuning: the number of components and the number of variables selected in each component. To determine the optimal number of components for sPLS-DA, we employed a 10-fold cross-validation repeated 10 times to assess prediction accuracy across different numbers of components. Given a matrix $\emptyset$ with known category labels for all samples (cells/spots), we randomly divide $\emptyset$ into 10 parts, using nine parts for training and the remaining one part for testing in turns. Assuming that the number of known categories is H, we set the maximum number of inferred components to $H-1$. We fit the PLS regression to the training set, and then apply PLS-DA for predicting the sample categories in the test set. For each new sample (cell/spot), the category with the highest prediction score is chosen as the prediction. Given a component number n from 1 to $H-1$, we calculated the average error rate over a total of 10 prediction rounds in the 10-fold cross-validation process. The

optimal number of components was estimated by the average error rates.

Upon the determination of the optimal number of components, the best feature gene set for each component can be obtained. Given a range for the number of feature genes in a component (*e.g.*, $n1 \sim n2$), sPLS-DA calculates the average error rate for each component restricted by the feature number to determine the optimal feature gene set for each component. To determine the optimal number of features, we proceeded in increments (default step size is 10), starting from 1 and continuing until n reaches the total number of genes. Meanwhile, the frequency of variables selected across folds during the repeated cross-validation is recorded. Since highly correlated variables are more likely to be chosen in each feature subset, we selected high-confidence genes that appear with a frequency higher than 90% in each component's feature subset, termed as sPLS genes.

Dimensionality reduction, clustering, and visualization by integrating multi-modal data

We employed the Seurat package for dimensionality reduction, clustering, and generation of the UMAP plot. For the imputed matrix $\emptyset^{+}$, we applied normalization using *LogNormalize* in function *NormalizeData*, and *vst* method in function *FindVariableFeatures* to identify HVGs. The data was then scaled using *ScaleData* and further reduced to two dimensions using *RunUMAP*. For sub-cluster identification within the PBMC data, we used the Louvain clustering based on shared nearest neighbors through *FindClusters*. Gene expression data for visualization were log-transformed. For spatial

clustering, we also utilized Louvain clustering using *FindClusters*, where the resolution parameter was adjusted for each dataset to match the number of morphological layers (Supplementary Table 11). In the clustering process of the gene expression matrix, top HVGs and top PCs were used, with the number depending on the dataset (Supplementary Table 11).

For visualization of a single modality with UMAP, the score matrix $T_{n\times K}$ obtained from PLS-DA was used as the input for the *umap* function in the R package uwot. For joint visualization of the matrix G and matrix $\emptyset$, we first separately computed the score matrices T_{rna} and T_{apa}, and then combined the two score matrices into a single matrix which serve as the input for the *umap* function. Assuming that the dimensionality of T_{rna} is $n\times K_1$ and the dimensionality of T_{apa} is $n\times K_2$, the dimensionality of the integrated matrix would be $n\times(K_1+K_2)$. Moreover, inspired by a previous supervised dimensionality analysis based on hybrid subset selection and linear discriminant analysis (HSS-LDA) [23], we also designed a semi-supervised dimensionality reduction and visualization workflow called PLS+UMAP. First, we adopted PLS to perform dimensionality reduction for the gene expression modality (G) and the APA modality ($\emptyset$ or $\emptyset^+$), respectively. Next, the PLS embeddings obtained from the two modalities were used to integrate information from both modalities for the UMAP plot. As a comparison, the gene expression matrix (G) and the RUD matrix ($\emptyset$ or $\emptyset^+$) were also used as input for Seurat's WNN-based multi-modal integration process to generate the UMAP plot.

Evaluation Metrics

Pearson's correlation coefficients (PCC) and silhouette score (SC) [24] were used to evaluate the performance of the imputation methods.

Since the APA profile of the imputed matrix $\emptyset^+$ should closely resemble that of the original matrix $\emptyset$, we used the PCC to evaluate the correlation between the two matrices. We calculated PCC of the RUD profiles (*i.e.*, φ values in the $\emptyset$ and $\emptyset^+$ matrix) of each cell before and after imputation. Using the average φ of cells in the same cell type in matrix $\emptyset$ (denoted as meta-φ) as the reference, we computed the PCC of φ values of each cell in the $\emptyset^+$ matrix with meta-φ.

SC measures separation between clusters without using known labels, which calculates the difference in distances between samples within the same cluster and to the nearest cluster. SC score ranges between -1 and 1. A higher SC score indicates that samples within the same cluster are closer, and samples from different clusters are further apart.

$$\mathrm{SC} = \frac{1}{\mathrm{c}}\sum_{\mathrm{i}}\left\{\frac{1}{\mathrm{n_i}}\sum_{\mathrm{x}\in \mathrm{V_i}}\frac{\mathrm{b(x)-a(x)}}{\mathrm{max[b(x),a(x)]}}\right\} \quad (5)$$

Here c is the number of clusters; $a(x)$ is the average distance between sample x and other samples within the same cluster; $b(x)$ is the average distance between sample x and all samples in the nearest cluster.

Note 1: evaluating the WNN-based imputation module in spvAPA

In spvAPA, WNN was used to merge K-nearest neighbor (KNN) graphs of the gene

expression modality and APA modality to infer nearest neighbor cells for imputation. To further assessed the effectiveness of WNN, we used a latest deep learning model called JAMIE (Joint Variational Autoencoders for multimodal Imputation and Embedding) [25] for multimodal integration and compared it with WNN. Results showed that WNN outperformed JAMIE in imputing APA signatures across all the seven datasets (Supplementary Fig. 2).

It is a common challenge in single-cell data where rare cell types are often underrepresented. Next, we examined the effect of the WNN-based imputation module on recovering missing entries in rare cell types. We used a random sampling strategy to simulate class imbalanced data. Briefly, given a dataset D with n cell types, we randomly selected 20 cells from the ith cell type to mimic a rare cell type and combined them with the other cells in D to form a new dataset D_{-i} that contained a rare cell type. Then D_{-i} was imputed with the WNN module of spvAPA to obtain an imputed matrix ${D_{-i}}^{+}$. Next, we calculated the PCC of cells in the rare cell type between D_{-i} and ${D_{-i}}^{+}$ (see Materials and Methods), denoted as PCC_{-i}. Meanwhile, the PCC of cells of the ith cell type can also be calculated between the raw data D and the imputed D^{+}, denoted as PCC_{i}. By comparing PCC_{-i} and PCC_{i}, we can examine the impact of cell number reduction on missing data imputation. For each cell type in D, the above process is repeated to obtain the PCC difference for each cell type. It can be seen that when the number of cells significantly decreases to the level of a rare cell type, the increase in PCC after imputation also decreases (Supplementary Fig. 3). Moreover, the degree of PCC reduction varies among different data. But for any rare cell

type simulated in this study, the PCC after imputation is still significantly higher than the PCC obtained from the raw data. This result indicates that the performance improvement of imputation on rare cell types is lower than that on normal cell types, but imputation always contributes to performance improvement.

The most important parameter in our WNN-based imputation model is the k value that determines the number of nearing neighbors. Here we test the impact of different k values (10, 15, 20) on APA matrix imputation using the SC-MOB data. Here we do not recommend using larger k values because single-cell transcriptomes often exhibit great heterogeneity and variability, and using too many neighboring cells may introduce excessive errors. The performance was evaluated by the PCC metric. The results show that PCC values have stable performance at different k values and are all relatively high (Supplementary Fig. 4a). Specifically, we also used sPLS-DA to perform APA feature selection on APA matrices imputed using the WNN module with different k values (Supplementary Fig. 4b). The results showed that the APA features obtained under different k values remained almost unchanged. These results indicate the robustness of the WNN method to different k values.

Next, we evaluated the computational efficiency of the WNN module on datasets with different number of genes and cells. The evaluation was conducted on an Ubuntu operating system with hardware configurations that include an Intel(R) Xeon(R) Gold 5218R (2.10 GHz, 80-core) CPU, 192GB of memory. As the number of cells or genes increases, the running time and memory usage generally increase linearly (Supplementary Fig. 5). The increase in the number of cells requires higher performance requirements than the increase

in the number of genes. Particularly, when the number of cells increases to 50000 or more, the demand for memory greatly increases (Supplementary Fig. 5a). However, even if the number of cells increases to 100,000, only about 4G memory is needed, which can be met by a general-purpose computer. These results demonstrate the scalability of the imputation module in spvAPA.

Note 2: using APA features to improve the dimensionality reduction visualization of scMOB data

We investigated whether the inclusion of APA features can further improve the dimensionality reduction visualization of scMOB data. First, we utilized sPLS-DA for dimensionality reduction, and identified components for the gene-cell expression matrix (G) and the APA matrix ($\emptyset^{+}$), respectively. Then the low dimensional embeddings were used as the input for UMAP visualization (called sPLS+UMAP). For comparison, we also conducted the standard dimensionality reduction provided in Seurat and then performed UMAP visualization (called Seurat+UMAP). Considering only the APA modality for visualization, the intra-class aggregation of neuronal cells (N) in the visualization generated by sPLS+UMAP is better than that in the visualization generated by the Seurat+UMAP; the Seurat+UMAP workflow led to neuronal cells (N) being divided into multiple dispersed clusters (Supplementary Fig. 12). The SC score also reflects better separation of cells types using sPLS+UMAP (0.607) than using Seurat+UMAP (0.449).

Upon close examination of the visualization results based on the two modalities (*i.e.*, matrix G and matrix $\emptyset^+$), we found several interesting insights. For instance, the UMAP plot based on the matrix G using sPLS+UMAP improved intra-cluster cohesion for myelinating oligodendrocytes (MyOligo) but failed to distinguish between mononuclear cells (Mono) and macrophages (MΦ). In contrast, the UMAP plot based on the matrix $\emptyset^+$ using sPLS+UMAP displayed superior intra-cluster cohesion for astrocytes (Astero) and was capable of distinguishing between mononuclear cells (Mono) and macrophages (MΦ). These results suggested that different modalities (*i.e.*, matrix G and matrix $\emptyset^+$) may have varying or complementary contributions to the differentiation of different cell types.

Note 3: integrating APA and gene modalities to enhance dimensionality reduction and visualization

It was found that matrix G and matrix $\emptyset^+$ each captured distinct sets of cell-type-specific genes (Figs. 6b-d and Supplementary Fig. 12), we then speculated whether integrating both matrices could enhance dimensionality reduction and visualization by leveraging the complementary information between different modalities. Accordingly, we designed a semi-supervised dimensionality reduction and visualization process using sPLS-DA and UMAP. We extracted components from matrix G and matrix $\emptyset^+$ with sPLS-DA, respectively. Then these components were concatenated and used as input for UMAP visualization. For comparison, we also performed Seurat's WNN to combine matrix G

and matrix $\emptyset^+$ for UMAP plot (WNN+UMAP). Although WNN+UMAP indeed improved the intra-cluster cohesion for OPC cells, it reduced the cohesion among neuronal cells (N) and microglia (MicroG) (Fig. 6e). In contrast, sPLS+UMAP not only excelled in distinguishing different cell types but also enhanced intra-cluster cohesion. For example, macrophages (MΦ) were clearly separated from monocytes (Mono) and microglia (MicroG) (Supplementary Fig. 12). In the visualization based solely on matrix G, macrophages (MΦ) situated very closely with monocytes (Mono) and microglia (MicroG). In contrast, using only the matrix $\emptyset^+$ for visualization, macrophages were more distinctly separated from the other two cell types. These results indicate that sPLS+UMAP visualization can integrate the complementary information from different modalities, leading to more accurately extracting latent biological differences associated with known labels. Similarly, when visualized using the matrix G alone, neuronalcells (N) were not well-separated from certain cell types but displayed relatively good intraclass aggregation. In contrast, when visualized using the matrix $\emptyset^+$ alone, neuronal cells were indistinguishable from other cell types either. However, upon integrating the matrix G and the matrix $\emptyset^+$ using sPLS+UMAP, not only does it maintain interclass separation, but it also improves the intraclass aggregation of neuronal cells. The SC score reached 0.68, which was higher than the values obtained using WNN+UMAP (Fig. 6e) or with single modality (Supplementary Fig. 12). These results demonstrated that the semi-supervised dimensionality reduction and visualization scheme of sPLS+UMAP provided in the spvAPA framework could effectively integrate multimodal data, facilitating dimensionality

reduction and visualization.

References

1. Tepe B, Hill MC, Pekarek BT et al. Single-cell RNA-seq of mouse olfactory bulb reveals cellular heterogeneity and activity-dependent molecular census of adult-born neurons, Cell Reports 2018;25:2689-2703.e2683.

2. Lukassen S, Bosch E, Ekici AB et al. Characterization of germ cell differentiation in the male mouse through single-cell RNA sequencing, Scientific Reports 2018;8:6521.

3. Chu L-F, Leng N, Zhang J et al. Single-cell RNA-seq reveals novel regulators of human embryonic stem cell differentiation to definitive endoderm, Genome Biology 2016;17:173.

4. Hao Y, Hao S, Andersen-Nissen E et al. Integrated analysis of multimodal single-cell data, Cell 2021;184:3573-3587.e3529.

5. Gao Y, Li L, Amos CI et al. Analysis of alternative polyadenylation from single-cell RNA-seq using scDaPars reveals cell subpopulations invisible to gene expression, Genome Research 2021;31:1856-1866.

6. Ståhl PL, Salmén F, Vickovic S et al. Visualization and analysis of gene expression in tissue sections by spatial transcriptomics, Science 2016;353:78-82.

7. Ji G, Tang Q, Zhu S et al. stAPAminer: Mining Spatial Patterns of Alternative Polyadenylation for Spatially Resolved Transcriptomic Studies, Genomics, Proteomics & Bioinformatics 2023:S1672022923000037.

8. Carrion SA, Michal JJ, Jiang Z. Alternative Transcripts Diversify Genome Function for Phenome Relevance to Health and Diseases, Genes (Basel) 2023;14.

9. Li Z, Chen X, Zhang X et al. Latent feature extraction with a prior-based self-attention framework for spatial transcriptomics, Genome Res 2023;33:1757-1773.

10. Liang Y, Shi G, Cai R et al. PROST: quantitative identification of spatially variable genes and domain detection in spatial transcriptomics, Nat Commun 2024;15:600.

11. Shi X, Yang Y, Ma X et al. Probabilistic cell/domain-type assignment of spatial transcriptomics data with SpatialAnno, Nucleic Acids Res 2023;51:e115.

12. Xu H, Fu H, Long Y et al. Unsupervised spatially embedded deep representation of spatial transcriptomics, Genome Medicine 2024;16:12.

13. Wong K, Navarro JF, Bergenstråhle L et al. ST Spot Detector: a web-based application

for automatic spot and tissue detection for spatial Transcriptomics image datasets, Bioinformatics 2018;34:1966-1968.

14. Rouillard AD, Gundersen GW, Fernandez NF et al. The harmonizome: a collection of processed datasets gathered to serve and mine knowledge about genes and proteins, Database (Oxford) 2016;2016.

15. Zheng GX, Terry JM, Belgrader P et al. Massively parallel digital transcriptional profiling of single cells, Nature Communications 2017;8:14049.

16. Haga Y, Sakamoto Y, Kajiya K et al. Whole-genome sequencing reveals the molecular implications of the stepwise progression of lung adenocarcinoma, Nat Commun 2023;14:8375.

17. Wu X, Liu T, Ye C et al. scAPAtrap: identification and quantification of alternative polyadenylation sites from single-cell RNA-seq data, Briefings in Bioinformatics 2021;22:bbaa273.

18. Patrick R, Humphreys DT, Janbandhu V et al. Sierra: discovery of differential transcript usage from polyA-captured single-cell RNA-seq data, Genome Biology 2020;21:167.

19. Ye W, Liu T, Fu H et al. movAPA: modeling and visualization of dynamics of alternative polyadenylation across biological samples, Bioinformatics 2021;37:2470-2472.

20. Stuart T, Butler A, Hoffman P et al. Comprehensive Integration of Single-Cell Data, Cell 2019;177:1888-1902.e1821.

21. Rohart F, Gautier B, Singh A et al. mixOmics: An R package for ‘omics feature selection and multiple data integration, PLOS Computational Biology 2017;13:e1005752.

22. Lê Cao K-A, Boitard S, Besse P. Sparse PLS discriminant analysis: biologically relevant feature selection and graphical displays for multiclass problems, BMC Bioinformatics 2011;12:253.

23. Amouzgar M, Glass DR, Baskar R et al. Supervised dimensionality reduction for exploration of single-cell data by HSS-LDA, Patterns 2022;3:100536.

24. Ferson S, Rohlf FJ, Koehn RK. Measuring shape variation of two-dimensional outlines, Systematic Biology 1985;34:59-68.

25. Cohen Kalafut N, Huang X, Wang D. Joint variational autoencoders for multimodal imputation and embedding, Nature Machine Intelligence 2023;5:631-642.
